# Cannabidiol improves muscular lipid profile by affecting the expression of fatty acid transporters and inhibiting de novo lipogenesis

**DOI:** 10.1038/s41598-023-30872-w

**Published:** 2023-03-06

**Authors:** Patrycja Bielawiec, Sylwia Dziemitko, Karolina Konstantynowicz-Nowicka, Adrian Chabowski, Janusz Dzięcioł, Ewa Harasim-Symbor

**Affiliations:** 1 Department of Physiology, Medical University of Bialystok, Bialystok, Poland; 2 Department of Human Anatomy, Medical University of Bialystok, Bialystok, Poland

**Keywords:** Diseases, Medical research

## Abstract

Obesity is one of the principal public health concerns leading to disturbances in glucose and lipid metabolism, which is a risk factor for several chronic diseases, including insulin resistance, type 2 diabetes mellitus, and cardiovascular diseases. In recent years, it turned out that cannabidiol (CBD) is a potential therapeutic agent in the treatment of obesity and its complications. Therefore, in the present study, we used CBD therapy (intraperitoneal injections in a dose of 10 mg/kg of body mass for 14 days) in a rat model of obesity induced by a high-fat diet (HFD). Gas–liquid chromatography and Western blotting were applied in order to determine the intramuscular lipid content and total expression of selected proteins in the white and red gastrocnemius muscle, respectively. Based on fatty acid composition, we calculated de novo lipogenesis ratio (16:0/18:2n-6), desaturation ratio (18:1n-9/18:0), and elongation ratios (18:0/16:0, 20:0/18:0, 22:0/20:0 and 24:0/22:0), in the selected lipid fractions. Two-week CBD administration significantly reduced the intramuscular fatty acids (FAs) accumulation and inhibited de novo lipogenesis in different lipid pools (in the free fatty acid, diacylglycerol, and triacylglycerol fractions) in both muscle types, which coincided with a decrease in the expression of membrane fatty acid transporters (fatty acid translocase, membrane-associated fatty acid binding protein, and fatty acid transport proteins 1 and 4). Moreover, CBD application profoundly improved the elongation and desaturation ratios, which was in line with downregulated expression of enzymes from the family of elongases and desaturases regardless of the metabolism presented by the muscle type. To our knowledge, this study is the first that outlines the novel effects of CBD action on skeletal muscle with different types of metabolism (oxidative vs. glycolytic).

## Introduction

Obesity is one of the most common medical concerns worldwide and its incidence is anticipated to rise alarmingly. The ongoing increase in obesity rates reflects lifestyle changes, particularly changes in nutrition. Although the reasons for its development are well known, including genetic factors, overnutrition, and a sedentary lifestyle, an effective prevention and treatment method is still being sought^1^. In the course of obesity, increased availability of fatty acids (FAs) in the diet leads to excessive storage of lipids in adipocytes and, subsequently, in other metabolically important tissues such as the liver, cardiac and skeletal muscle^2^. Several lines of evidence have shown that intramuscular lipid accumulation, especially in the fractions of triacylglycerol (TAG), diacylglycerol (DAG), and ceramide (CER), is due to increased transmembrane transport of long-chain fatty acids (LCFAs)^3^. Moreover, numerous studies indicate the detrimental effect of some lipid molecules (DAG, CER), occurring in increased amounts intracellularly, on the downstream insulin signal transduction pathway^4,5^. In turn, this results in dysregulation of glucose and lipid metabolism, and thus disturbed cellular and, subsequently, whole-body energy homeostasis^6^. A shift in the energy balance is the major cause of metabolic comorbidities, including insulin resistance (IR), type 2 diabetes mellitus (T2DM), and nonalcoholic fatty liver disease (NAFLD), which together account for a large number of obesity-related deaths^7,8^.

The LCFAs transport across the plasma membrane of cells is of fundamental importance as these compounds perform a variety of functions, among others, they are the building blocks of membranes, fuel for energy supply, and play a role in different signaling pathways^9,10^. They can be derived directly from the diet or they may be synthesized de novo through lipogenesis^11^. A large number of studies performed in the last two decades has shown that FAs are transported across the plasma membrane of skeletal muscle not only by passive diffusion but mainly with the use of highly specialized protein transporters including fatty acid translocase (CD36), membrane-associated fatty acid binding protein (FABPpm) and fatty acid transport proteins 1 and 4 (FATP-1,4) (Fig. 9)^12,13^. Subsequently, inside the myocyte, exogenous FAs derived from the diet along with palmitic acid (16:0), which is the primary end product of de novo lipogenesis, undergo further elongation processes producing a variety of long-chain and very long-chain fatty acids, including saturated fatty acids (SFAs), monounsaturated fatty acids (MUFAs) and polyunsaturated fatty acids (PUFAs)^14^. The enzymes involved in the elongation process are referred to as the elongation of very long-chain fatty acids proteins (ELOVL)^15^. There are 7 different elongase subtypes (ELOVL1-7), which show different substrate selectivity and mediate a wide range of specific elongation reactions^16^. To date, it has been established that ELOVL1,3,6 and 7 are selective for SFA and MUFA, while ELOVL2,4 and 5 preferentially utilize PUFA^17^. Moreover, functional diversity of lipids is possible not only due to the variability of their chain length but also the degree of their unsaturation, which is conditioned by the acyl-coenzyme A (CoA) desaturases. This family of enzymes introduces a double bond in a specific position on the acyl chain of LCFA, and so far two subgroups of desaturases have been identified, namely stearoyl-coenzyme A (CoA) desaturases (SCDs) and fatty acid desaturases (FADS)^14,17^. SCD1 is a central lipogenic enzyme that plays a pivotal role in the regulation of MUFA biosynthesis^17^. It catalyzes the formation of palmitoleate (16:1n-7) and oleate (18:1n-9), from SFA palmitate (16:0) and stearate (18:0), respectively^18^. However, when it comes to the second subgroup, the ∆-5 (FADS1) and ∆-6 desaturase (FADS2) are the key enzymes in the metabolism of n-3 and n-6 PUFA, enabling the formation of long-chain metabolites from α-linoleic acid (ALA) and linoleic acid (LA)^19^. Disturbances in the elongation and desaturation process may disrupt the FAs balance and, thereby, proper cellular functioning and metabolic homeostasis, which may be implicated in various diseases related to obesity occurrence^20^.

For several years, cannabidiol (CBD) has been in the spotlight as a potential therapeutic agent in the treatment of obesity. Among phytocannabinoids isolated from the *Cannabis sativa* plant (more than 120 compounds), CBD exhibits a great safety profile and lack of psychoactive properties^21,22^. Many studies have demonstrated CBD’s therapeutic potential due to its anti-inflammatory, antioxidant, anxiolytic, anticonvulsant, and neuroprotective properties^23,24^. In recent years, CBD has been shown to exert its effect on the endogenous system of signaling lipids called the endocannabinoid system (ECS), the definition of which has evolved into the extended ECS or the endocannabinoidome (eCBome)^25^. The eCBome encompassing endocannabinoids (e.g., N-arachidonoylethanolamine-anandamide (AEA) and 2-arachidonoylglycerol (2-AG)) and numerous long-chain fatty acid-derived congeners, their metabolic enzymes, and the receptors of these lipid compounds, including cannabinoid receptors (such as CB_1_ and CB_2_), orphan G protein-coupled receptors (such as GPR55 and GPR18), thermosensitive transient receptor potential (TRP) channels (such as TRPV1), and peroxisome proliferator-activated receptors (PPARs such as PPARα and PPARγ)^26,27^. So far, numerous studies have shown that CBD exhibits a weak affinity for CB_1_ and CB_2_ receptors, whereas it modulates to a much greater extent other molecular targets^28,29^. Furthermore, it has also been shown that CBD alters the eCBome tone by increasing the concentration of AEA, due to fatty acid amide hydrolase (FAAH) inhibition^30^. Research in the last decade has significantly increased our knowledge of the complexity of CBD’s action, however, its potential mechanisms for obesity treatment are not yet fully revealed. Therefore, the main purpose of this study was to investigate the influence of CBD on the expression of selected fatty acid transporters (FAT/CD36, FABPpm, and FATP-1,4), the content of various lipid fractions (free fatty acid (FFA), DAG, TAG, and phospholipid (PL)), de novo lipogenesis as well as stearoyl CoA-desaturase (SCD1) activity, and elongation ratios of selected FAs in the mentioned above lipid fractions in the white and red skeletal muscle (*musculus gastrocnemius*) of rats with obesity induced by a high-fat diet.

## Results

### Effect of chronic CBD administration on the total expression of proteins involved in fatty acid uptake in rats subjected to standard and high-fat diets

**Figure 1:**
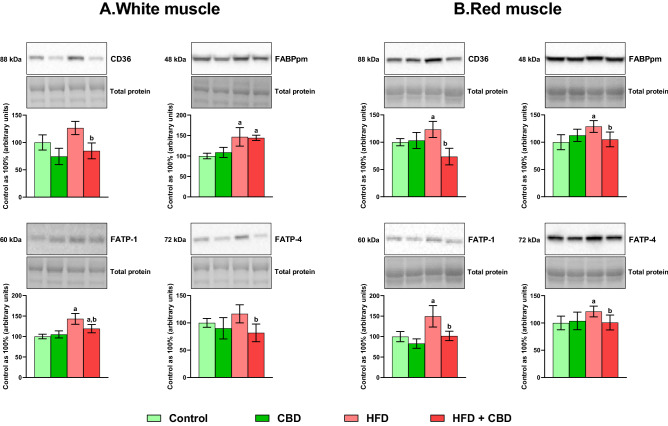
The total expression of proteins involved in fatty acid uptake: fatty acid translocase (CD36), fatty acid binding protein (FABPpm) and fatty acid transport protein 1 and 4 (FATP-1 and -4), in the white and red gastrocnemius muscle. The total expressions of the abovementioned proteins are presented as percentage differences compared to the control group which was set as 100%. The data are expressed as mean values ± SD, n = 6 in each group; ^a^*p* < 0.05 indicates a significant difference: the control group vs. the examined group; ^b^*p* < 0.05 indicates a significant difference: HFD vs. HFD + CBD.

Our experiment revealed that induction of obesity by high-fat diet feeding significantly raised the levels of both FABPpm and FATP-1 in the white skeletal muscle (+ 46.73% and + 32.40%, respectively, *p* < 0.05; Fig. 1A) compared to the control group. However, in the red skeletal muscle, levels of all examined transporters were considerably elevated after HFD feeding (CD36: + 23.38%, FABPpm: + 28.77%, FATP-1: + 49.54%, FATP-4: + 21.01%, vs. the control group, *p* < 0.05; Fig. 1B). Moreover, the intramuscular expression of fatty acid transporters, i.e., CD36, FABPpm as well as FATP-1 and FATP-4, were significantly changed in the group fed HFD after the introduction of CBD (FABPpm: + 44.31%, FATP-1: + 19.21%, vs. the control group, FATP 1: 9.96%, FATP-4: − 29.82%, CD36: − 33.11%, vs. the HFD group, *p* < 0.05; Fig. 1A) in the white skeletal muscle. Concomitantly, CBD injections to animals fed HFD caused a substantial decrease in the expression of each transporter in the red gastrocnemius muscle (CD36: − 40.35%, FABPpm: − 18.37%, FATP-1: − 32.01%, FATP-4: − 16.69%, *p* < 0.05; Fig. 1B) compared to the HFD group.

### Effect of chronic CBD administration on total free fatty acid, diacylglycerol, triacylglycerol and phospholipid content in rats subjected to standard and high-fat diets

**Figure 2:**
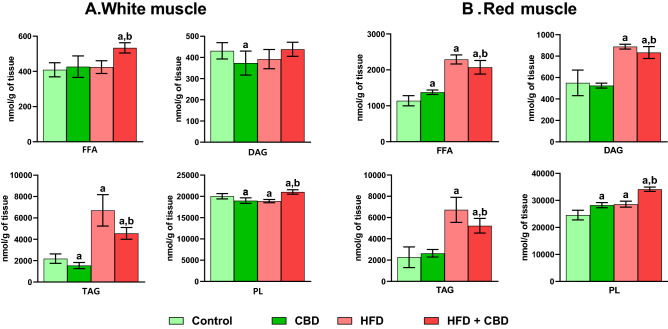
Total free fatty acid (FFA), diacylglycerol (DAG), triacylglycerol (TAG) and phospholipid (PL) content in the white and red gastrocnemius muscle. Values are expressed in nmol per mg of tissue. The data are expressed as mean values ± SD, n = 10 in each group; ^a^*p* < 0.05 indicates a significant difference: the control group vs. the examined group; ^b^*p* < 0.05 indicates a significant difference: HFD vs. HFD + CBD.

The study presented a significant increase in the pool of FFA in the group fed HFD after two-week CBD treatment (+ 30.46%, vs. the control group; + 25.79%, vs. the HFD group, *p* < 0.05; Fig. 2A) in the white gastrocnemius muscle. DAG fraction was markedly reduced after the administration of CBD (− 13.36%, vs. the control group, *p* < 0.05; Fig. 2A), whereas in the TAG pool not only the decrease in the same group occurred (− 29.51%, vs. the control group, *p* < 0.05), but also pronounced changes in the HFD (+ 206.58%, vs. the control group, *p* < 0.05) and HFD + CBD groups were observed (+ 108.15%, vs. the control group; − 32.11%, vs. the HFD group, *p* < 0.05; Fig. 2A) in the white skeletal muscle. PL fraction was substantially diminished in the groups treated with CBD as well as fed HFD (− 5.19% and − 5.70%, vs. the control group, respectively, *p* < 0.05; Fig. 2A) with simultaneous elevation in the HFD + CBD group (+ 5.09%, vs. the control group; + 11.44%, vs. the HFD group, *p* < 0.05; Fig. 2A) in the white gastrocnemius muscle. In contrast, in the red skeletal muscle the above fraction was increased in CBD, HFD as well as HFD + CBD groups (+ 14.90%, + 16.39%, + 38.86%, respectively, *p* < 0.05; Fig. 1B) in comparison with control animals. Moreover, in the same tissue, significant elevation also occurred in the rats fed HFD and treated with CBD (+ 19.31%, *p* < 0.05; Fig. 2B) compared to rats fed HFD. Additionally, the content of FFA was considerably heightened in all examined groups (CBD: + 20.81%, HFD: + 101.02%, HFD + CBD: + 81.88%, respectively, *p* < 0.05; Fig. 1B) compared to the control group, and substantially diminished in the HFD + CBD group (− 9.52%, *p* < 0.05; Fig. 1B) compared to the HFD group. The levels of DAG and TAG fractions in the red gastrocnemius muscle were markedly elevated in animals fed HFD as well as fed HFD and administered with CBD (DAG: + 61.42%, + 51.41%, TAG: + 196.71%, 130.68%, respectively, *p* < 0.05; Fig. 2B) in comparison to control animals. In contrast, the content of both fractions was lessened in rats receiving HFD and CBD (DAG: − 6.20% and TAG: − 22.25%, *p* < 0.05; Fig. 2B) compared to the rats receiving only HFD (see Supplementary File S1 for the total fatty acid composition of the individual lipid fractions in the white and red gastrocnemius muscle).

### Effect of chronic CBD administration on intramuscular de novo lipogenesis ratio in the free fatty acid, diacylglycerol, triacylglycerol and phospholipid fractions in rats subjected to standard and high-fat diets

**Figure 3:**
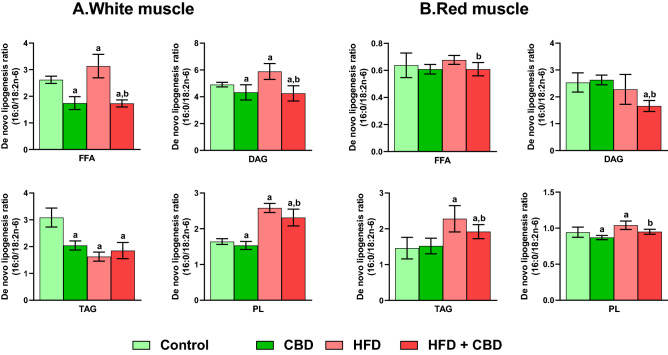
Intramuscular de novo lipogenesis in the free fatty acid (FFA), diacylglycerol (DAG), triacylglycerol (TAG) and phospholipid (PL) fractions in the white and red gastrocnemius muscle. The data are expressed as mean values ± SD, n = 10 in each group; ^a^*p* < 0.05 indicates a significant difference: the control group vs. the examined group; ^b^*p* < 0.05 indicates a significant difference: HFD vs. HFD + CBD.

In the white skeletal muscle, the de novo lipogenesis ratio in the FFA and DAG fractions was elevated in the HFD (+ 21.54%, + 20.02%, vs. the control group, respectively, *p* < 0.05; Fig. 3A) but diminished in the CBD group (− 33.48%, − 11.80%, vs. the control group, respectively, p < 0.05; Fig. 3A) as well as in HFD group administered with CBD (− 33.92%, − 13.32% vs. the control group, respectively; − 45.63%, − 27.78%, vs. the HFD group, respectively, *p* < 0.05; Fig. 3A). Interestingly, in the red skeletal muscle significant changes in FFA and DAG pools were observed only in rats receiving a diet rich in fatty acids and CBD injections (FFA: − 10.16%, DAG: − 34.57%, vs. the control group, respectively; DAG: − 27.14%, vs. the HFD group, *p* < 0.05; Fig. 3B). The 16:0/18:2n-6 ratio in TAG fraction was markedly diminished in each examined group (CBD: -33.75%, HFD: − 47.20%, HFD + CBD: − 39.92%, vs. the control group, *p* < 0.05; Fig. 3A) In the white gastrocnemius muscle, while in the red skeletal muscle it was substantially changed in HFD (+ 56.22%, vs. the control group, *p* < 0.05) and HFD + CBD groups (+ 31.57%, vs. the control group; − 15.78%, vs. the HFD group, *p* < 0.05; Fig. 3B). Introduction of CBD to animals fed standard and rich in fatty acid diet led to significant changes of 16:0/18:2n-6 ratio in PL pool in the red muscle (CBD: − 7.81% vs. the control group, HFD + CBD: − 8.67%, vs. HFD group, *p* < 0.05; Fig. 3B) as well as in the white muscle (CBD: − 6.64%, HFD + CBD: + 40.94% vs. the control group, − 10.39%, vs. the HFD group, *p* < 0.05; Fig. 3A). Concomitantly, in both types of muscle tissue in the above-mentioned fraction, we observed a substantial rise after induction of obesity by high-fat diet feeding (white muscle: + 57.28%, Fig. 3A; red muscle: + 10.22% *p* < 0.05; Fig. 3B) compared to the control group.

### Effect of chronic CBD administration on intramuscular stearoyl-coenzyme A desaturase 1 level in the free fatty acid, diacylglycerol, triacylglycerol and phospholipid fractions in rats subjected to standard and high-fat diets

**Figure 4:**
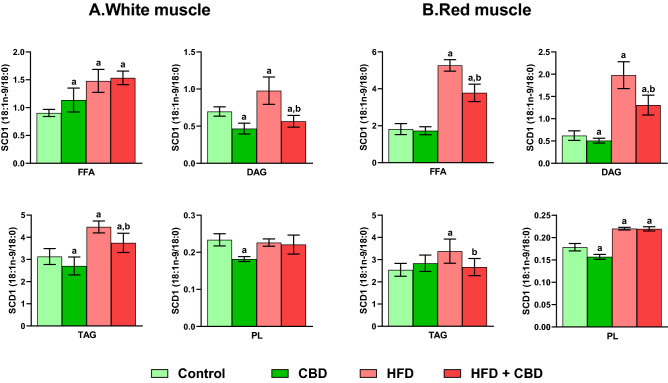
Intramuscular stearoyl-coenzyme A desaturase 1 (SCD1) activity in the free fatty acid (FFA), diacylglycerol (DAG), triacylglycerol (TAG) and phospholipid (PL) fractions in the white and red gastrocnemius muscle. The data are expressed as mean values ± SD, n = 10 in each group; ^a^*p* < 0.05 indicates a significant difference: the control group vs. the examined group; ^b^*p* < 0.05 indicates a significant difference: HFD vs. HFD + CBD.

As shown in Fig. 4A, we noticed pronounced changes in the 18:1n-9/18:0 ratio in FFA (CBD: + 25.66%, HFD: + 63.57%, HFD + CBD: 69.67%, vs. the control group, *p* < 0.05), DAG (CBD: − 32.97%, HFD: + 40.37%, HFD + CBD: − 18.91%, vs. the control group, − 42.23%, vs. the HFD group, *p* < 0.05) and TAG fractions (CBD: − 13.61%, HFD: + 42.67%, HFD + CBD: + 19.71%, vs. the control group, − 16.10%, vs. the HFD group, *p* < 0.05;), whereas in the PL pool changes occurred only in the group treated with CBD (− 22.16%, vs. the control group, *p* < 0.05) in white gastrocnemius muscle. In red gastrocnemius muscle, administration of CBD to animals fed standard chow led to the diminishment of the 18:1n-9/18:0 ratio only in DAG and PL fractions (− 18.15% and − 12.18%, vs. the control group, respectively, *p* < 0.05; Fig. 4B), however, CBD treatment in animals on high-fat chow led to significant changes in each examined lipid fraction (FFA: + 107.56%, vs. the control group, − 28.25%, vs. the HFD; DAG: + 110.65%, vs. the control group, − 33.99%, vs. the HFD; TAG: − 21.08% vs. the HFD; PL: + 22.94%, vs. the control group, *p* < 0.05; Fig. 4B). In the same type of tissue, we also observed substantial elevation in SCD1 in rodents receiving HFD (FFA: + 189.28%; DAG: + 219.12%; TAG: + 32.89%; PL: + 23.18%, *p* < 0.05; Fig. 4B) compared to the rodents on the standard diet.

### Effect of chronic CBD administration on intramuscular elongation ratios in the free fatty acid, diacylglycerol, triacylglycerol and phospholipid fractions in rats subjected to standard and high-fat diets

**Figure 5:**
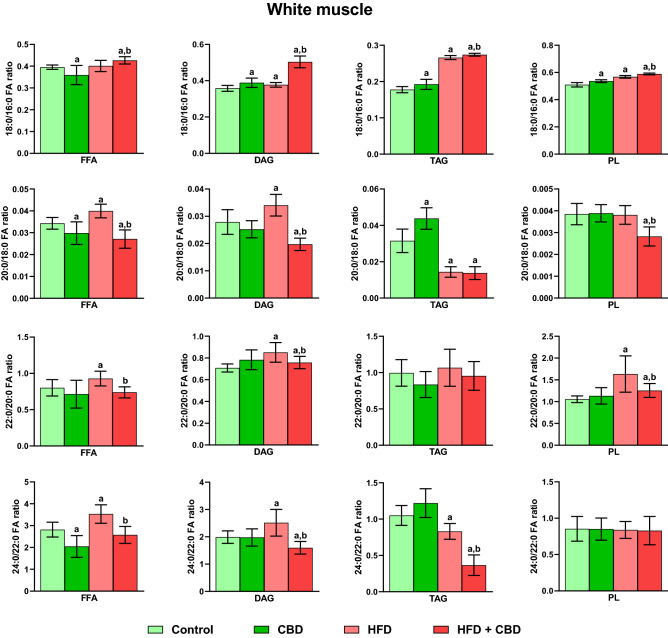
Intramuscular 18:0/16:0, 20:0/18:0, 22:0/20:0 and 24:0/22:0 elongation ratios in the free fatty acid (FFA), diacylglycerol (DAG), triacylglycerol (TAG) and phospholipid (PL) fractions in the white gastrocnemius muscle. The data are expressed as mean values ± SD, n = 10 in each group; ^a^*p* < 0.05 indicates a significant difference: the control group vs. the examined group; ^b^*p* < 0.05 indicates a significant difference: HFD vs. HFD + CBD.

**Figure 6:**
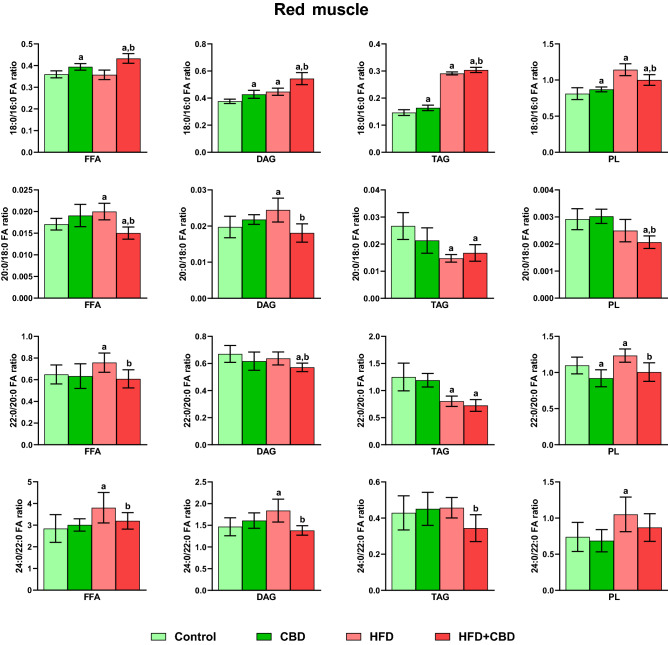
Intramuscular 18:0/16:0, 20:0/18:0, 22:0/20:0 and 24:0/22:0 elongation ratios in the free fatty acid (FFA), diacylglycerol (DAG), triacylglycerol (TAG) and phospholipid (PL) fractions in the red gastrocnemius muscle. The data are expressed as mean values ± SD, n = 10 in each group; ^a^*p* < 0.05 indicates a significant difference: the control group vs. the examined group; ^b^*p* < 0.05 indicates a significant difference: HFD vs. HFD + CBD.

Our experiment demonstrated that the elongation 18:0/16:0 ratio was significantly altered after the introduction of CBD to rodents on standard and rich in fatty acids diet in FFA (CBD: − 9.14%, HFD + CBD: + 7.89%, vs. the control group and + 6.30%, vs. the HFD group, *p* < 0.05; Fig. 5), DAG (CBD: + 8.57%, CBD + HFD: + 40.55%, vs. the control group and + 33.43%, vs. the HFD group, *p* < 0.05; Fig. 5), TAG (CBD: + 8.08%, HFD + CBD: + 54.10%, *p* < 0.05, vs. the control group and + 3.0%, vs. the HFD group, *p* < 0.05; Fig. 5) and PL pools (CBD: + 5.28%, HFD + CBD: + 15.66%, vs. the control group and + 3.66%, vs. the HFD group, *p* < 0.05; Fig. 5) in the white skeletal muscle. Additionally, induction of obesity by a diet rich in fats led to a substantial rise in the 18:0/16:0 elongation ratio in selected lipid fractions: DAG, TAG as well as PL (+ 5.34%, + 49.64% and + 11.58%, respectively, *p* < 0.05; Fig. 5) compared to the control group in the white skeletal muscle. In the same type of tissue, the elongation 20:0/18:0 ratio was significantly changed in examined fractions of FFA (CBD: − 13.14%, HFD: + 16.45%, HFD + CBD: − 20.93%, vs. the control group and − 32.10%, vs. the HFD group, *p* < 0.05; Fig. 5), DAG (HFD: + 25.08%, HFD + CBD: − 29.32%, vs. the control group and − 43.49%, vs. the HFD group, *p* < 0.05; Fig. 5), TAG (CBD: + 38.77%, HFD: − 54.92%, HFD + CBD: − 52.26%, vs. the control group, *p* < 0.05; Fig. 5) and PL (HFD + CBD: − 27.22%, vs. the control group and − 26.49%, vs. the HFD group, *p* < 0.05; Fig. 5). We also observed that the elongation 22:0/20:0 ratio was considerably heightened in the high-fat diet fed rats (FFA: + 15.93%, DAG: + 20.93% and PL: + 54.94%, *p* < 0.05; Fig. 5) compared to rats fed standard chow in white gastrocnemius muscle. Administration of CBD in the HFD group led to the elevation of the above-mentioned ratio in DAG and PL pools (+ 6.98% and + 19.08%, respectively, *p* < 0.05; Fig. 5) in comparison to the control, yet it caused a substantial diminishment in the FFA, DAG and PL fractions (− 20.40%, − 11.54%, − 23.15%, respectively, *p* < 0.05; Fig. 5) in comparison to the HFD group in white gastrocnemius muscle. Finally, the elongation 24:0/22:0 ratio in the white skeletal muscle was markedly altered in 3 out of 4 examined lipid fractions: FFA (CBD: − 27.31%, HFD: + 25.38%, vs. the control group, HFD + CBD: − 27.13%, vs. the HFD group, *p* < 0.05; Fig. 5), DAG (HFD: + 26.41%, HFD + CBD: − 19.71%, vs. the control group and − 36.48%, vs. the HFD group, *p* < 0.05; Fig. 5) and TAG (HFD: − 20.87%, HFD + CBD: − 65.16%, vs. the control group and − 55.97%, vs. the HFD group, *p* < 0.05; Fig. 5). Regarding the red gastrocnemius muscle, the same ratio was substantially increased by prolonged administration of fatty acids in selected lipid fractions: FFA, DAG as well as PL (+ 33.74%, + 21.40% and + 42.12%, respectively, *p* < 0.05; Fig. 6) in comparison with the control group. Concomitantly, the introduction of CBD in the HFD group led to the substantial diminishment of this ratio in FFA, DAG and TAG fractions (− 15.95%, -24.93% and − 24.74%, respectively, *p* < 0.05; Fig. 6) in comparison with the HFD group. We noticed that elongation 18:0/16:0 ratio in the red muscle after administration of CBD to animals on standard and rich in fats chow was heightened in FFA (CBD: + 9.64%, HFD + CBD: + 20.34%, vs. the control group and + 21.08%, vs. the HFD group, p < 0.05; Fig. 6), DAG (CBD: + 13.58%, HFD + CBD: + 44.36%, vs. the control group and + 21.62%, vs. the HFD group, p < 0.05; Fig. 6), TAG (CBD: + 11.72%, HFD + CBD: + 107.40%, vs. the control group and + 3.02%, *p* < 0.05, vs. the HFD group; Fig. 6) and PL fractions (CBD: + 7.34%, HFD + CBD: + 23.32%, vs. the control group, p < 0.05; Fig. 6), whereas lessened only in PL fractions (HFD + CBD: − 12.50%, vs. the HFD group, *p* < 0.05; Fig. 6). The described elongation ratio was markedly increased after HFD feeding in DAG, TAG as well as PL (+ 18.70, + 101.32% and + 40.93%, vs. the control group, respectively, *p* < 0.05; Fig. 6) pools in the red skeletal muscle. As shown in Fig. 6, in the same type of tissue, the experimental induction of the obesity by HFD altered the elongation 20:0/18:0 (FFA: + 17.30%, DAG: + 21.08%, TAG: − 44.21%, vs. the control group, *p* < 0.05) as well as 22:0/20:0 ratio (FFA: + 16.73%, TAG: − 35.79%, PL: + 12.46%, vs. the control group, *p* < 0.05) in examined lipid pools. Moreover, CBD injections to animals fed HFD caused a substantial decrease of both elongation 20:0/18:0 and 22:0/20:0 ratios in all examined lipid fractions (20:0/18:0: FFA: − 11.95%, TAG: − 37.19%, PL: − 29.12%, vs. the control group, PL: − 17.05%, DAG: − 26.03%, FFA: − 24.93, vs. the HFD group; 22:0/20:0: DAG: − 14.75%, TAG: − 41.98%, vs. the control group, FFA: − 19.70%, DAG: − 10.26%, PL: − 18.50%, vs. the HFD group, *p* < 0.05; Fig. 6) in red gastrocnemius muscle. Additionally, the two-week CBD treatment of animals receiving standard chow considerably influenced only the 22:0/20:0 ratio in PL fraction (− 16.08%, vs. the control group, *p* < 0.05; Fig. 6) in the red skeletal muscle.

### Effect of chronic CBD administration on the total expression of proteins involved in fatty acid synthesis and metabolism in rats subjected to standard and high-fat diets

**Figure 7:**
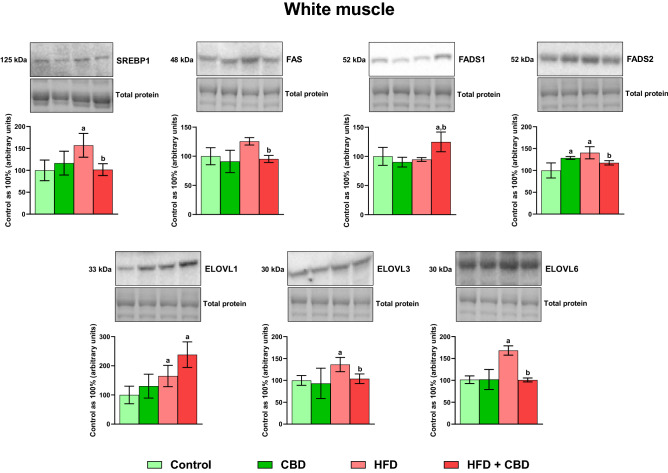
The total expression of proteins involved in the fatty acid synthesis and metabolism: sterol regulatory element-binding protein 1 (SREBP1), fatty acid synthase (FAS), fatty acid desaturase 1 and 2 (FADS1 and FADS2) as well as fatty acid elongase 1, 3, and 6 (ELOVL1, ELOVL3, and ELOVL6) in the white gastrocnemius muscle. The total expressions of the abovementioned proteins are presented as percentage differences compared to the control group which was set as 100%. The data are expressed as mean values ± SD, n = 6 in each group; ^a^*p* < 0.05 indicates a significant difference: the control group vs. the examined group; ^b^*p* < 0.05 indicates a significant difference: HFD vs. HFD + CBD.

**Figure 8:**
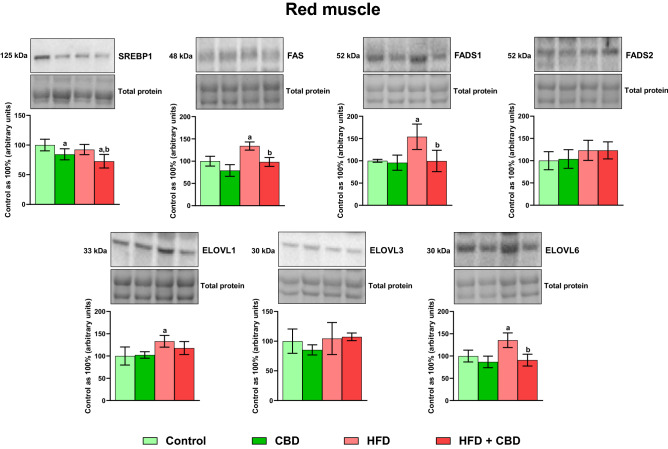
The total expression of proteins involved in the fatty acid synthesis and metabolism: sterol regulatory element-binding protein 1 (SREBP1), fatty acid synthase (FAS), fatty acid desaturase 1 and 2 (FADS1 and FADS2) as well as fatty acid elongase 1, 3, and 6 (ELOVL1, ELOVL3, and ELOVL6) in the red gastrocnemius muscle. The total expressions of the abovementioned proteins are presented as percentage differences compared to the control group which was set as 100%. The data are expressed as mean values ± SD, n = 6 in each group; ^a^*p* < 0.05 indicates a significant difference: the control group vs. the examined group; ^b^*p* < 0.05 indicates a significant difference: HFD vs. HFD + CBD.

In our experiment, we revealed that a high-fat chow administration caused a significant increase in the total expression of sterol regulatory element-binding protein 1 (SREBP1) in the white gastrocnemius muscle (+ 53.74%, *p* < 0.05, vs. the control group; Fig. 7), which was further reduced by the CBD implementation (− 10.82%, *p* < 0.05, vs. the HFD group; Fig. 7). On the contrary, in the red skeletal muscle, induction of obesity by high-fat feeding resulted in a slight downregulation of SREBP1 expression in comparison with the control rats. However, two-week CBD treatment substantially decreased the SREBP1 expression in standard chow-fed control rats (− 15.66%, *p* < 0.05, vs. the control group) and in the lipid oversupply conditions in relation to the untreated control and HFD group alone (− 27.26% and − 21.23%, *p* < 0.05, respectively; Fig. 8). As shown in Fig. 7, in the white muscle homogenates, we observed decreased fatty acid synthase (FAS) expression after the introduction of CBD to rats on diet rich in fatty acids (− 24.13%, *p* < 0.05, vs. the HFD group). Moreover, induction of obesity by high-fat diet feeding markedly altered the expression of each examined isoform of ELOVL, namely ELOVL1, ELOVL3, and ELOVL6 (+ 65.04%, + 36.19%, − 37.34% and + 65.81%, vs. the control group, respectively, *p* < 0.05; Fig. 7) in the white skeletal tissue. Additionally, CBD injections to animals on high-fat chow resulted in an increase in ELOVL1 expression in the white skeletal muscle (+ 138.15%, *p* < 0.05; Fig. 7) compared to the group receiving standard chow, whereas the content of ELOVL3 and ELOVL6 was substantially diminished in the described group (− 23.80% and − 40.04%, respectively, *p* < 0.05; Fig. 7) in comparison with the HFD group. Concomitantly, in red skeletal muscle induction of obesity by a diet rich in fatty acids significantly enhanced only ELOVL1 and ELOVL6 (+ 33.09%, + 35.54%, vs. the control group, respectively, *p* < 0.05; Fig. 8). Additionally, administration of CBD to the HFD group only diminished the expression of ELOVL6 isoform (− 32.98%, vs. the HFD group, *p* < 0.05; Fig. 8) in the red skeletal muscle. Concomitantly, we observed elevated intramuscular expression of FADS1 only in animals receiving HFD and treated with CBD (+ 24.81%, vs. the control group; + 31.92%, vs. the HFD, *p* < 0.05; Fig. 7) in the white gastrocnemius muscle, whereas FADS2 expression in the same type of tissue was increased in CBD group (+ 28.47%, vs. the control group, *p* < 0.05; Fig. 7) and HFD group (+ 40.47%, vs. the control group, *p* < 0.05; Fig. 7), yet diminished in HFD + CBD group (− 16.45%, vs. the HFD group, *p* < 0.05; Fig. 7). In contrast, in the red skeletal muscle, we did not notice any pronounced changes in total FADS2 expression, however, induction of obesity by high-fat chow feeding significantly heightened intramuscular expression of FADS1 as well as FAS (+ 54.06% and + 34.12%, vs. the control group, respectively, *p* < 0.05; Fig. 8). Importantly, CBD treatment during the course of high-fat feeding considerably reduced FADS1 and FAS expression (− 35.38% and − 26.74%, vs. the HFD group, respectively, *p* < 0.05; Fig. 8) in the red gastrocnemius muscle.

## Discussion

In this study, we demonstrated for the first time that CBD, a non-psychotropic plant-origin cannabinoid, reduces the expression of LCFA transport proteins and intramuscular lipid accumulation with simultaneous inhibition of de novo lipogenesis in skeletal muscle of rats in the course of obesity induced by a high-fat diet. Moreover, we have shown that CBD under these conditions is a modulator of the elongation and desaturation processes, which positively influenced FAs metabolism.

In our study, as we expected, the induction of obesity by feeding rats a HFD significantly increased the expression of all examined LCFAs transporters (CD36, FABPpm, FATP-1, and FATP-4) in the red gastrocnemius muscle, while in the white skeletal muscle only expression of FABPpm and FATP-1 was enhanced. These differences are probably related to the metabolism presented by these types of muscles, where white skeletal muscle exhibits glycolytic metabolism and red skeletal muscle exhibits oxidative metabolism^31^. In addition, we found that CBD appears to function as a regulator of the expression of LCFAs transport proteins, as its two-week administration markedly attenuated the total expression of specific LCFAs transporters, i.e., CD36, FABPpm, FATP-1, and FATP-4 in both types of skeletal muscles. In line with that, we observed similar effects of CBD action on the expression of fatty acid protein carriers in the myocardial tissue, which we described in our previous work^32^. It is worth emphasizing, that CBD had the greatest effect on the muscular expression of CD36, which is believed to play a pivotal role in maintaining cellular lipid homeostasis^12,33^. Numerous studies have shown that complete deletion or downregulation of CD36 expression significantly reduces the uptake of LCFAs, thereby causing changes in fatty acid metabolism, especially concerning fatty acid oxidation, which is important in the working heart and skeletal muscles^34^. Other researchers have also shown that the overexpression of CD36 caused by lipid oversupply, increases the influx of LCFAs into the cell, exceeding the oxidative capacity of mitochondria, which in turn results in the storage of excessive amounts of FAs in the TAG fraction and its further conversion to more metabolically active lipid metabolites (i.e., DAG, CER), which are known to inhibit the insulin signaling pathway, promoting the development of IR^35^. Furthermore, recent studies have shown the unexpected properties of CD36 to promote de novo lipogenesis in hepatocytes by regulating SREBP1 processing^36^. Zeng et al. found that CD36 directly interacted with an insulin-induced gene (INSIG) to attenuate its inhibitory effects on SREBP1 proteolysis. Increased processing of SREBP1 upregulated the expression of de novo lipogenesis enzymes such as acetyl-CoA carboxylase α (ACCα) and FAS, which resulted in elevated hepatic lipid accumulation^36^. Considering the above, CBD downregulates protein-mediated LCFAs transport in myocytes of obese rats, which is desired therapeutic effect in future clinical studies.

It is well known that in the course of obesity there is the deposition of lipids in non-adipose tissues such as skeletal muscle^9^. In our research, we confirmed it by noting a significant increase in intramuscular accumulation of FAs in various lipid fractions including FFA, DAG, TAG, and PL in both muscle types in fatty acids oversupply conditions, which is in line with a significant upregulation of LCFAs transport proteins expression in either white and red skeletal muscle. However, we have observed a much greater FAs accumulation in the red gastrocnemius muscle in all examined lipid fractions. It is consistent with the metabolism shown by this type of muscle tissue, since the red muscle fibers use FAs as the primary source of energy in the oxidation process, while white skeletal muscles obtain energy through the glycolytic pathway^31^. Moreover, it is also associated with a more pronounced expression of membrane LCFAs transport proteins in the red skeletal muscle^31^. Particular attention should be paid to the increased accumulation of lipids not only in the TAG fraction but also in the fraction of DAG, which was observed in our study in the red skeletal muscle. DAG is one of the prime candidates among the lipid derivatives responsible for the development of IR. Many studies have shown that it disrupts the insulin signaling pathway by activating both classic and atypical protein kinase C (PKC), which dephosphorylates insulin receptor substrate 1 (IRS1) and thus blocks further downstream insulin signaling, thereby reducing insulin-stimulated glucose uptake^37,38^. In contrast, TAG is considered to be a relatively safe fraction that does not exhibit intracellular lipotoxic activity, because both the activation and inhibition of TAG intramuscular hydrolysis do not significantly affect the mitochondrial function and cellular insulin sensitivity^39^. Accordingly, the herein study demonstrated that in animals receiving a HFD after a two-week administration of CBD, we noticed a remarkable decrease in TAG content in the white gastrocnemius muscle, whereas in the red skeletal muscle CBD injections significantly reduced lipid accumulation in FFA, DAG, and TAG fractions. This is in line with the results obtained by Silvestri et al.^22^ where they showed that CBD dose-dependently inhibits TAG accumulation in hepatocytes and 3T3-L1 adipocytes treated with oleic acid (OA). Interestingly, only in the case of the PL fraction, we observed that chronic CBD treatment caused a pronounced increase in the content of accumulated lipids in the rats fed a HFD in both white and red skeletal muscle. We can hypothesize that CBD enhances the incorporation of FAs into the PL fraction and significantly influences the composition of the above-mentioned fraction in favor of n-3 PUFA, as we demonstrated in our previous work^40^. Concomitantly, in our experiment, we reported intensified muscular de novo lipogenesis in rats subjected to high-fat feeding in both white and red gastrocnemius muscle as indicated by increased lipogenic indexes. De novo lipogenesis is a complex metabolic pathway in which excess carbohydrates are converted into FAs, which further are esterified and stored as TAG fraction^41^.This also underlines the importance of de novo lipogenesis process in the skeletal muscle, especially in the state of IR related to obesity^42^.

Furthermore, recent research has shown the relationship between ECS and FAs metabolism. Osei-Hyiaman et al.^43^ demonstrated that hepatic CB_1_ activation results in enhanced de novo fatty acid synthesis via the induction of the lipogenic transcription factor SREBP-1c and its target enzymes including FAS. Interestingly, in our previous research^6^, we revealed that CBD downregulated muscle CB_1_ receptor expression, however, we can assume that CBD in skeletal muscle affects FAS expression through a different molecular mechanism than in the liver, due to the fact that both tissues differ significantly in terms of metabolic processes occurring in them. In addition, CB_1_ has also been shown to be associated with SCD1 activity. A study conducted by Liu et al. revealed that various overlapping mechanisms contribute to the development of IR, more specifically stimulation of the ECS by CB_1_ receptor activation in the course of high-fat diet-induced obesity promotes the activity of SCD1, an enzyme that catalyzes the biosynthesis of MUFA^44,45^. The same study also revealed that endogenous MUFAs (palmitoleic and oleic acid) act as fatty acid amide hydrolase (FAAH) inhibitors leading to a reduction in AEA degradation and an elevation in its concentration, which stimulates the ECS. Furthermore, elevated levels of MUFA activate CB_1_ receptors that promote de novo lipogenesis by inducing the expression of lipogenic genes, including SCD1, forming a positive feedback loop^45^. This is in accordance with our studies, which also showed a pronounced upregulation of SCD1 activity in rats fed a HFD in all examined lipid fractions in the red and white gastrocnemius muscle, only with the exception of the PL fraction in the glycolytic type of muscle. The two-week administration of CBD significantly reduced the level of SCD1 in the DAG and TAG fractions in the white muscle, again affecting the red muscle to a greater extent, where it decreased SCD1 activity in the FFA, DAG, and TAG lipid pools. Based on the above data, we may suspect that the effects of CBD are related to the inhibition of the endocannabinoids action at the CB_1_ receptor level since CBD has been well-described as a negative allosteric modulator of this cannabinoid receptor^46^. Additionally, in our experimental model, we examined the muscle expression of fatty acid desaturase 1 (FADS1; also known as delta-5 desaturase (∆5D)) and fatty acid desaturase 2 (FADS2; referred as delta-6 desaturase (∆6D)), enzymes involved in the double bond introduction at delta ∆5 and ∆6 positions of PUFA chain, respectively^19^. Our analysis, after treatment of HFD-fed rats with CBD, indicated substantially decreased expression of FADS1 in the red gastrocnemius muscle and FADS2 in the white skeletal muscle. We believe that this may be considered as a cell protection mechanism since Yashiro et al. reported that FADS1 inhibition alleviates IR and reduce body weight in a HFD-induced obese (DIO) mouse model^47^. Moreover, FADS1 catalyzes the production of arachidonic acid (20:4, AA), which is a precursor of pro-inflammatory eicosanoids, therefore, its accumulation promotes the synthesis of AA-derived mediators that contribute to the development of chronic inflammation related to obesity^48^. Similarly, a growing number of studies demonstrated a positive correlation between increased FADS2 expression and IR as well as TD2M occurrence, whereas FADS2 inhibition resulted in resistance to HFD-induced obesity in a mouse model^49,50^. Intriguingly, in the white gastrocnemius muscle we also noticed a substantial rise in the FADS1 expression in the HFD + CBD group. This is in line with the results from our previous publication, where we discovered that the most pronounced changes considering the AA and eicosapentaenoic acid (EPA) content (the synthesis of which is controlled by FADS1), were observed in the same experimental group in different lipid fractions in the white skeletal muscle^40^. However, a stronger protective effect of CBD treatment was observed in the red gastrocnemius muscle, where it caused a significant shift in the n-6/n-3 PUFA balance towards the anti-inflammatory n-3 PUFA in examined lipid fractions, which we can associate with the metabolism exhibited by this muscle type. This taken altogether, can lead to the presumption that CBD in conditions of elevated FAs consumption inhibits de novo fatty acids synthesis and reduces their intracellular accumulation in the skeletal muscle in obesity.

**Figure 9:**
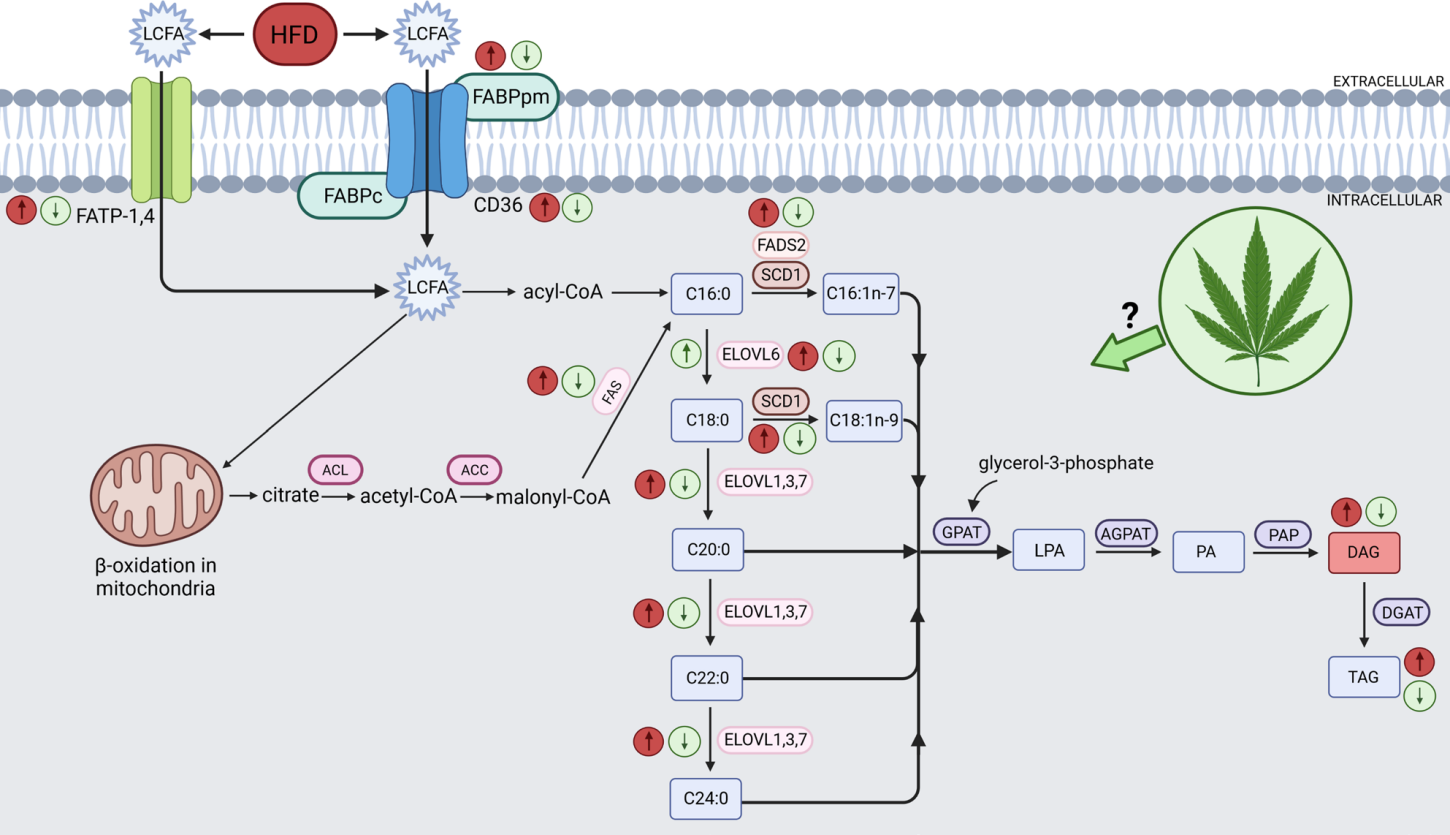
Effects of a high-fat diet (HFD) and two-week cannabidiol (CBD) administration on the fatty acid transport proteins expression, de novo lipogenesis, elongation and desaturation processes in rat myocytes. ↑, increase; ↓, decrease; red arrow inside the circle indicates the effects of seven weeks of high-fat diet feeding; green arrow inside the circle indicates the effects of two weeks of CBD treatment in HFD-fed rats; long-chain fatty acids (LCFA); fatty acid transport protein 1 and 4 (FATP-1 and -4); cytoplasmic and membrane-associated fatty acid binding protein (FABPc, FABPpm); fatty acid translocase (CD36); palmitic acid (C16:0); palmitoleic acid (C16:1n-7); stearic acid (C18:0); oleic acid (C18:1n-9); arachidic acid (C20:0); behenic acid (C22:0); lignoceric acid (C24:0); ATP citrate lyase (ACL); acetyl-CoA carboxylase (ACC); fatty acid synthase (FAS); fatty acid desaturase 2 (FADS2); stearoyl-coenzyme A (SCD1); elongation of very long-chain fatty acids proteins 1,3,6 and 7 (ELOVL1,3,6 and 7); glycerol-3-phosphate acyltransferase (GPAT); lysophosphatidic acid (LPA); 1-acylglycerol-3-phosphate-O-acyltransferase (AGPAT); phosphatidic acid (PA); phosphatidic acid phosphatase (PAP); diacylglycerol (DAG); diglyceride acyltransferase (DGAT); triacylglycerol (TAG). Figure was created in BioRender by the authors.

In addition, we examined for the first time the effect of CBD action on the expression of the elongase family members, where we revealed substantial alternations in the elongation process in both white and red skeletal muscle. Overall, LCFAs supplied with the diet and formed during de novo lipogenesis (mainly 16:0; palmitic acid) can be elongated by adding two carbon units to the carboxyl end of LCFAs, using malonyl-CoA and fatty acyl-CoA as substrates. This process is catalyzed by the ELOVLs (Fig. 9). Our findings indicate that a HFD increases elongation of 16∶0 to stearic acid (18∶0) in the DAG, TAG, and PL lipid fractions in the skeletal muscle of rats regardless of the “presented metabolism”. Intriguingly, CBD treatment in high-fat feeding markedly elevated the 18:0/16:0 FA elongation ratio in the following fractions: FFA, DAG, and TAG in both muscle types. We also observed similar effects of CBD administration in rats subjected to the standard diet, where it enhanced a ratio of 18:0/16:0 elongation in the DAG, TAG, and PL fractions in the white gastrocnemius muscle, and in all examined lipid fractions in the red gastrocnemius muscle. Possibly, increased elongation of 16:0 by treatment with CBD can help prevent a buildup of 16∶0 in favor of 18∶0 in the skeletal muscle of obese rats, whichcan be considered as a cell protection mechanism. Accordingly, the increased elongation of palmitic acid to stearic acid reflects a significant rise in the expression of the ELOVL6 enzyme in rats fed a HFD in both muscle types, which was subsequently diminished by CBD. This is worth emphasizing since Matsuzaka et al.^51^ showed that mice with *Elovl6* gene deletion were resistant to the development of diet-induced IR despite the presence of obesity, suggesting that inhibition of elongase 6 may represent a novel approach to treat IR. In the case of further elongation of the FAs chain, our results show that the supply of a HFD to rats significantly increased the elongation ratio of stearic acid (18:0) to arachidic acid (20:0), arachidic acid to behenic acid (22:0) and behenic acid to lignoceric acid (24:0) in the FFA, DAG and PL lipid pools in either muscle with glycolytic and oxidative metabolism, whereas injections with CBD caused a significant reduction of the above-mentioned indexes. In addition, our research showed that the TAG fraction was affected to a lesser extent, and surprisingly chronic exposure to a HFD resulted in a decrease in the 20:0/18:0 and 24:0/22:0 indexes in the white gastrocnemius muscle, while in the red skeletal muscle, 20:0/18:0 and 22:0/20:0 ratios were reduced. However, the implementation of CBD treatment significantly diminished the elongation of 22:0 to 24:0 in both muscle types as well. Moreover, in our study, we investigated the muscular expressions of ELOVL1 and 3, the enzymes that elongate mostly SFA and MUFA providing precursors for the synthesis of sphingolipids^52^. The total expression of ELOVL1 and ELOVL3 was significantly enhanced in both white and red gastrocnemius muscles of the rats subjected to a HFD, which is consistent with results obtained by Kozawa et al.^53^, and coincides with the increase in elongation ratios determined in our study (18:0/16:0, 20:0/18:0, 22:0/20:0 and 24:0/22:0). Although, in the oxidative fibers, we did observe only a trend towards downregulation in the muscular expression of elongase 1 and 3 in the HFD + CBD group, while in the case of anaerobic fibers, two-week CBD administration significantly reduced total ELOVL3 expression and surprisingly increased the expression of ELOVL1 in the same group of animals. Collectively, these findings implicate that lipid oversupply to skeletal muscle, results in increased elongation of SFA chains, which, take part in further metabolic transformations, and intensify the accumulation of compounds such as CER and DAG, since most SFAs in mammals are found to be components of sphingolipids family^52^. These active biomolecules, as indicated by a substantial body of evidence, impair the insulin-signaling cascade by inhibiting insulin-stimulated phosphorylation of protein kinase B (Akt) and glycogen synthase kinase 3 (GSK3β), and subsequent GLUT4 translocation from the intracellular compartments to the plasma membrane, which partially was also showed in our previous work^6,54^. Collectively, the interactions between the above processes are quite complex, and examined enzymes are involved in different synthetic pathways that can alter the levels of various LCFAs pools in response to distinct factors, which is the basis for further research examining the exact mechanisms of CBD action.

## Conclusions

Taken altogether, the study presented herein has demonstrated the novel effects of CBD action as a potential drug to combat obesity and its complications. Under normal conditions, there appear to be closely coordinated regulations between LCFAs intracellular transport, de novo lipogenesis, elongation, and desaturation. Whereas the excess delivery of LCFA significantly affects these processes, leading to dysregulation of lipid metabolism, which contributes to the development of IR. However, the results obtained here indicate that CBD is involved in the regulation of intramuscular FAs uptake and thus reduces the accumulation of lipids in the skeletal muscles and has a positive effect on their further metabolic fate. Hence, CBD properties appear to be an attractive therapeutic strategy for obesity treatment.

## Materials and methods

### Animals and experimental protocol

All experimental procedures were conducted on male Wistar rats, initially weighing 70–100 g. The animals were delivered from the Center for Experimental Medicine of the Medical University of Bialystok, Poland. Rats were kept in approved animal holding facilities (22 ± 2 °C with a reverse light–dark cycle of 12/12 h) and had free access to water and standard rodent chow (Labofeed B, Animal Feed Manufacturer “Morawski”, Kcynia, Poland). After seven days of acclimatization, rodents were randomly divided into four experimental groups, each consisting of 10 animals: (1) control group—receiving a basal rodent diet (12.4 kcal% fat, 57.1 kcal% carbohydrates, and 30.5 kcal% protein), (2) CBD group—receiving a basal rodent diet and treated with CBD, (3) HFD group—receiving a rodent diet rich in fatty acids (60 kcal% fat, 20 kcal% carbohydrates, and 20 kcal% protein (cat. no.: D12492, Research Diets Inc., New Brunswick, NJ, USA^55^)) and (4) HFD + CBD group—receiving a rodent diet rich in fatty acids and treated with CBD (Table 1). According to the described groups, animals were fed an appropriate diet for seven weeks. Simultaneously, intraperitoneal (i.p.) injections of CBD or its vehicle were given to the animals, starting from the fifth week. Daily injections with synthetic CBD (10 mg/kg, purity ≥ 99%; THC Pharm GmbH, Frankfurt, Germany) or its solvent for control and HFD groups (3:1:16, ethanol, Tween-80, and 0.9% NaCl) lasted for two weeks^56,57^. Then, at the end of the 7th week of the experiment and 24 h after the last dose of CBD or its vehicle, rats from all experimental groups were anesthetized i.p. with pentobarbital (80 mg/kg). Additionally, the body weight of each animal was monitored during the whole experiment and we reported substantially increased body mass in rats subjected to the high fat feeding, however two-week CBD treatment did not significantly affect the body weight in rats fed both the standard chow and HFD (the results were published in our previous publication^32^). Muscle samples (musculus gastrocnemius) were excised, then immediately frozen with aluminum tongs precooled in liquid nitrogen and stored at − 80 °C for subsequent analysis. To obtain plasma samples, a puncture through the inferior vena cava was performed, blood was collected to heparinized test tubes and centrifuged. The experimental protocol was approved by the local Animal Ethics Committee in Olsztyn under license number 71/2018 and all methods were carried out in accordance with relevant guidelines and regulations. Methods are reported in accordance with ARRIVE guidelines.

**Table 1:** The nutritional composition of the high-fat diet (HFD).

Class description	Ingredient	HFD (g)
Protein	Casein, lactic, 30 mesh Cystine, L	200.00 3.00
Carbohydrate	Lodex 10 Sucrose, fine granulated	125.00 72.80
Fiber	Solka floc, FCC200	50.00
Fat	Soybean oil, USP Lard	25.00 245.00
Mineral	S10026B	50.00
Vitamin	Choline bitartrate V10001C	2.00 1.00

### Skeletal muscle lipid analysis

Gas–liquid chromatography (GLC) was performed to analyze intramuscular lipid fractions, namely DAG, TAG, PL, and FFA fractions. The pulverization of frozen muscle samples in mortar precooled in liquid nitrogen was followed by overnight extraction in chloroform–methanol solution (2:1, vol/vol) according to the method of Folch^58^, with the addition of butylated hydroxytoluene as an antioxidant and heptadecanoic acid as an internal standard. Afterward, the samples were centrifuged and the lower layer was collected for subsequent analysis. The above-mentioned lipid fractions were separated by thin-layer chromatography (TLC) on silica gel plates (Silica Plate 60, 0.25 mm; Merck, Darmstadt, Germany), using a heptane/isopropyl ether/acetic acid (60:40:3, vol/vol/vol) as a resolving solution. Visualization of dried silica plates under ultraviolet light enabled the identification of target lipid fractions. Thereafter, gel bands corresponding to selected lipid fractions were scrapped and eluted. DAG, TAG, PL, and FFA fractions were eluted in appropriate solutions and the organic phase was transmethylated in a 14% boron trifluoride-methanol (BF3) solution. Samples with the addition of hexane were examined by a Hewlett Packard 5890 Series II Gas Chromatograph (Agilent Technologies, CA, USA) containing a capillary column (50 m × 0.25 mm inner diameter) and a flame ionization detector—HP-INNOWax. Individual fatty acids in each fraction were identified. Based on a sum of the particular fatty acid species content in each target fraction, the concentration of total DAG, TAG, PL, and FFA was calculated and expressed in nanomoles per gram of tissue. The de novo lipogenesis ratio was calculated as palmitic/linoleic acid (16:0/18:2n-6) ratio; SCD1 was measured as oleic/stearic acid (18:1n-9/18:0) ratio; elongation was estimated as stearic/palmitic acid (18:0/16:0) ratio, arachidic/stearic acid (20:0/18:0) ratio, behenic/arachidic acid (22:0/20:0) ratio as well as lignoceric/behenic acid (24:0/22:0) ratio.

### Western blotting

To examine selected protein expression, a Western Blotting procedure was performed. Obtained muscle samples were homogenized in radioimmunoprecipitation assay (RIPA) buffer containing a cocktail of protease and phosphatase inhibitors (Roche Diagnostics GmbH, Manheim, Germany). Then, the bicinchoninic acid method (BCA) with bovine serum albumin (BSA) as a standard was performed in order to measure total protein concentration. Thereafter, the homogenates were reconstituted in Laemmli buffer (Bio-Rad, Hercules, CA, USA) and applied on CriterionTM TGX Stain-Free precast gels (Bio-Rad, Hercules, CA, USA). After the electrophoresis, the separated proteins were transferred onto polyvinylidene fluoride (PVDF) (semi-dry transfer) or nitrocellulose membranes (wet transfer). The next step was blocking membranes in the Tris-buffered saline with Tween-20 (TBST) and 5% non-fat dry milk or 5% BSA, subsequently, the membranes were incubated overnight at 4 °C with selected primary antibodies: CD36 (sc-7309, Santa Cruz Biotechnology, Inc., Dallas, TX, USA), FABPpm (ab180162, Abcam, Cambridge, United Kingdom), FATP-1 (sc-25541, Santa Cruz Biotechnology, Inc., Dallas, TX, USA), FATP-4 (sc-5834, Santa Cruz Biotechnology, Inc., Dallas, TX, USA), SREBP1 (sc-367, Santa Cruz Biotechnology, Inc., Dallas, TX, USA), FAS (3180S, Cell Signalling Technology, Danvers, MA, USA), FADS1 (ab126706, Abcam, Cambridge, United Kingdom), FADS2 (ab232898, Abcam, Cambridge, United Kingdom), ELOVL1 (ab230634, Abcam, Cambridge, United Kingdom), ELOVL3 (sc-54878, Santa Cruz Biotechnology, Inc., Dallas, TX, USA) and ELOVL6 (sc-385127, Santa Cruz Biotechnology, Inc., Dallas, TX, USA). The membranes were then incubated with a secondary antibody conjugated with horseradish peroxidase (HRP) (Cell Signaling Technology, Danvers, MA, USA). The addition of the appropriate substrate for HRP (Clarity Western ECL Substrate; Bio-Rad, Hercules, CA, USA) was followed by the visualization of protein bands using a ChemiDoc visualization system (Image Laboratory Software Version 6.0.1; Bio-Rad, Warsaw, Poland). Stain-free gels and the total protein normalization method (Bio-Rad, Hercules, CA, USA) were applied to quantify the expression of the examined proteins (see Supplementary File S2). The total expressions of the abovementioned proteins were presented as percentage differences compared to the control group which was set as 100%, and are based on six independent determinations.

### Statistical analysis

All data obtained from the experiment are expressed as mean values ± standard deviation (SD). Statistical analysis was performed with the use of GraphPad Prism version 7.0 for Windows (GraphPad Software, La Jolla, CA, USA). The normality of the result distribution was checked using the Shapiro–Wilk test and the homogeneity of the variance with the use of Bartlett’s test. Then, a two-way test ANOVA and an appropriate post hoc test were carried out to indicate statistical differences. Values *p* < 0.05 were considered significant for all results.

## Supplementary Information

Supplementary Information 1.

Supplementary Information 2.

## Data Availability

The raw datasets used during the current study are available from the corresponding author on reasonable request.
