# Supplementary material for: Cannabidiol improves muscular lipid profile by affecting the expression of fatty acid transporters and inhibiting de novo lipogenesis
Source: Sci Rep. 2023 Mar 6;13:3694. doi: 10.1038/s41598-023-30872-w (PMC9988888; doi:10.1038/s41598-023-30872-w)
Supplement: Supplementary file 1 — Supplementary Information 1. [file 41598_2023_30872_MOESM1_ESM.pdf]

White skeletal muscle

Supplementary Table S1. Individual fatty acids content in the FFA fraction (nmol/g of wet tissue) in the white gastrocnemius muscle in the control (standard diet) and high-fat diet (HFD) groups after two-week cannabidiol (CBD) treatment.

Fatty acid	Control	CBD	HFD	HFD + CBD
**14:0**	22.14 ± 2.87	21.27 ± 4.11	27.92 ± 7.48 [a]	20.26 ± 2.37 [b]
**16:0**	159.96 ± 23.43	150.37 ± 33.49	145.74 ± 26.68	161.80 ± 14.35
**16:1n7**	12.10 ± 1.32	13.28 ± 2.77	9.76 ±1.79 [a]	14.38 ± 2.41 [b]
**18:0**	66.20 ± 11.97	62.01 ± 15.08	55.13 ± 8.15 [a]	71.79 ± 9.24 [b]
**18:1n9**	57.72 ± 10.08	69.67 ± 20.50	74.60 ± 13.50 [a]	119.42 ± 10.02 [a,b]
**20:0**	2.40 ± 0.35	1.93 ± 0.39 [a]	2.13 ± 0.39	1.93 ± 0.27 [a]
**22:0**	1.94 ± 0.38	1.89 ± 0.43	1.79 ± 0.42	1.52 ± 0.18 [a]
**24:0**	6.17 ± 1.31	4.20 ± 0.81 [a]	4.99 ± 0.84 [a]	3.79 ± 0.90 [a,b]
**24:1n9**	1.38 ± 0.27	1.43 ± 0.25	1.35 ± 0.14	0.97 ± 0.17 [a,b]

The data are expressed as mean values ± SD, n = 10 in each group. $^{a}p < 0.05$ indicates a significant difference: the control group vs. the examined group in the white gastrocnemius muscle; $^{b}p < 0.05$ indicates a significant difference: HFD vs. HFD + CBD in the white gastrocnemius muscle. 14:0 (myristic acid); 16:0 (palmitic acid); 16:1n7 (palmitoleic acid); 18:0 (stearic acid); 18:1n9 (oleic acid); 20:0 (arachidic acid); 22:0 (behenic acid); 24:0 (lignoceric acid); 24:1n9 (nervonic acid).

The content of polyunsaturated fatty acids (PUFA): 18:2n6 (linoleic acid), 18:3n3 (α-linolenic acid), 20:4n6 (arachidonic acid), 20:5n3 (eicosapentaenoic acid) and 20:6n3 (docosahexaenoic acid) in the FFA fraction in the white gastrocnemius muscle was published in our previous publication: Bielawiec et al. (doi: 10.3390/nu13051603).

Supplementary Table S2. Individual fatty acids content in the DAG fraction (nmol/g of wet tissue) in the white gastrocnemius muscle in the control (standard diet) and high-fat diet (HFD) groups after two-week cannabidiol (CBD) treatment.

Fatty acid	Control	CBD	HFD	HFD + CBD
**14:0**	27.45 ± 7.59	33.69 ± 9.78	23.27 ± 5.98	28.56 ± 4.33
**16:0**	217.01 ± 34.90	169.69 ± 34.26 [a]	156.80 ± 23.56 [a]	173.11 ± 23.86 [a]
**16:1n7**	8.73 ± 0.82	6.41 ± 1.24 [a]	6.26 ± 1.58 [a]	5.57 [a] ± 1.07
**18:0**	81.82 ± 12.93	68.40 ± 11.85 [a]	56.81 ± 6.84 [a]	90.97 ± 14.38 [b]
**18:1n9**	56.23 ± 10.02	31.46 ± 4.80 [a]	53.88 ± 9.58	53.34 ± 9.15
**20:0**	2.29 ± 0.24	1.85 ± 0.46 [a]	2.31 ± 0.63	1.78 ± 0.26 [a,b]
**22:0**	1.59 ± 0.20	1.50 ± 0.30	1.57 ± 0.51	1.53 ± 0.24
**24:0**	3.18 ± 0.32	2.92 ± 0.48	3.09 ± 0.42	2.51 ± 0.39 [a,b]
**24:1n9**	1.11 ± 0.24	0.85 ± 0.16 [a]	1.19 ± 0.35	0.89 ± 0.13 [a,b]

The data are expressed as mean values ± SD, n = 10 in each group. $^{a}p < 0.05$ indicates a significant difference: the control group vs. the examined group in the white gastrocnemius muscle; $^{b}p < 0.05$ indicates a significant difference: HFD vs. HFD + CBD in the white gastrocnemius muscle. 14:0 (myristic acid); 16:0 (palmitic acid); 16:1n7 (palmitoleic acid);

18:0 (stearic acid); 18:1n9 (oleic acid); 20:0 (arachidic acid); 22:0 (behenic acid); 24:0 (lignoceric acid); 24:1n9 (nervonic acid).

The content of polyunsaturated fatty acids (PUFA): 18:2n6 (linoleic acid), 18:3n3 (α-linolenic acid), 20:4n6 (arachidonic acid), 20:5n3 (eicosapentaenoic acid) and 20:6n3 (docosahexaenoic acid) in the FFA fraction in the white gastrocnemius muscle was published in our previous publication: Bielawiec et al. (doi: 10.3390/nu13051603).

Supplementary Table S3. Individual fatty acids content in the TAG fraction (nmol/g of wet tissue) in the white gastrocnemius muscle in the control (standard diet) and high-fat diet (HFD) groups after two-week cannabidiol (CBD) treatment.

Fatty acid	Control	CBD	HFD	HFD + CBD
**14:0**	70.18 ± 12.62	44.34 ± 9.81 [a]	120.34 ± 21.34 [a]	90.55 ± 14.11 [a,b]
**16:0**	910.56 ± 168.78	605.37 ± 112.01 [a]	1726.44 ± 588.80[a]	1596.05 ± 214.60 [a]
**16:1n7**	130.19 ± 19.27	106.15 ± 38.12	148.07 ± 48.62	130.50 ± 43.64
**18:0**	166.28 ±31.11	114.17 ± 18.22 [a]	489.56 ± 158.25 [a]	419.55 ± 71.98 [a,b]
**18:1n9**	523.38 ± 130.35	337.36 ± 83.95 [a]	2418.58± 653.68[a]	1634.38 ± 300.92 [a,b]
**20:0**	5.24 ± 1.46	4.81 ± 0.55	6.15 ± 1.34	5.71 ± 1.08
**22:0**	5.37 ± 1.49	4.15 ± 1.12 [a]	5.61 ± 1.59	6.50 ± 1.46
**24:0**	5.50 ± 1.44	5.64 ± 0.70	4.21 ± 1.03 [a]	2.18 ± 0.90 [a,b]
**24:1n9**	3.83 ± 1.22	2.64 ± 0.80 [a]	2.91 ± 1.14	3.15 ± 1.22

The data are expressed as mean values ± SD, n = 10 in each group. [a]$p < 0.05$ indicates a significant difference: the control group vs. the examined group in the white gastrocnemius muscle; [b]$p < 0.05$ indicates a significant difference: HFD vs. HFD + CBD in the white gastrocnemius muscle. 14:0 (myristic acid); 16:0 (palmitic acid); 16:1n7 (palmitoleic acid); 18:0 (stearic acid); 18:1n9 (oleic acid); 20:0 (arachidic acid); 22:0 (behenic acid); 24:0 (lignoceric acid); 24:1n9 (nervonic acid).
The content of polyunsaturated fatty acids (PUFA): 18:2n6 (linoleic acid), 18:3n3 (α-linolenic acid), 20:4n6 (arachidonic acid), 20:5n3 (eicosapentaenoic acid) and 20:6n3 (docosahexaenoic acid) in the FFA fraction in the white gastrocnemius muscle was published in our previous publication: Bielawiec et al. (doi: 10.3390/nu13051603).

Supplementary Table S4. Individual fatty acids content in the PL fraction (nmol/g of wet tissue) in the white gastrocnemius muscle in the control (standard diet) and high-fat diet (HFD) groups after two-week cannabidiol (CBD) treatment.

Fatty acid	Control	CBD	HFD	HFD + CBD
**14:0**	82.48 ± 9.72	55.26 ± 7.50 [a]	67.81 ± 7.20 [a]	57.11 ± 5.99 [a,b]
**16:0**	6319.50 ± 336.78	5608.23 ± 1109.50 [a]	5819.05 ± 236.40 [a]	5908.39 ± 1395.59
**16:1n7**	151.99 ± 21.81	127.52 ± 12.86 [a]	87.13 ± 6.31 [a]	96.00 ± 11.85 [a,b]
**18:0**	3216.55 ± 157.16	3196.03 ± 166.30	3292.10 ± 142.70 [a]	3602.94 ± 491.54
**18:1n9**	739.71 ± 48.41	580.55 ± 31.32 [a]	738.69 ± 26.00	853.98 ± 95.24 [a,b]
**20:0**	12.80 ± 1.70	12.43 ± 1.37	11.85 ± 2.18	11.41 ± 2.03
**22:0**	15.30 ± 4.48	15.07 ± 4.42	17.10 ± 3.47	15.18 ± 3.56
**24:0**	14.11 ± 2.75	12.94 ± 1.98	13.79 ± 2.72	12.87 ± 3.53
**24:1n9**	12.75 ± 1.97	13.84 ± 2.48	11.87 ± 2.25	9.93 ± 2.09 [a,b]

The data are expressed as mean values ± SD, n = 10 in each group. [a]p < 0.05 indicates a significant difference: the control group vs. the examined group in the white gastrocnemius muscle; [b]p < 0.05 indicates a significant difference: HFD vs. HFD + CBD in the white gastrocnemius muscle. 14:0 (myristic acid); 16:0 (palmitic acid); 16:1n7 (palmitoleic acid); 18:0 (stearic acid); 18:1n9 (oleic acid); 20:0 (arachidic acid); 22:0 (behenic acid); 24:0 (lignoceric acid); 24:1n9 (nervonic acid).

The content of polyunsaturated fatty acids (PUFA): 18:2n6 (linoleic acid), 18:3n3 (α-linolenic acid), 20:4n6 (arachidonic acid), 20:5n3 (eicosapentaenoic acid) and 20:6n3 (docosahexaenoic acid) in the FFA fraction in the white gastrocnemius muscle was published in our previous publication: Bielawiec et al. (doi: 10.3390/nu13051603).

Red skeletal muscle

Supplementary Table S5. Individual fatty acids content in the FFA fraction (nmol/g of wet tissue) in the red gastrocnemius muscle in the control (standard diet) and high-fat diet (HFD) groups after two-week cannabidiol (CBD) treatment.

Fatty acid	Control	CBD	HFD	HFD + CBD
**14:0**	29.62 ± 4.29	31.88 ± 4.35	42.44 ± 4.52 [a]	32.65 ± 5.52 [b]
**16:0**	268.41 ± 48.79	315.23 ± 14.52 [a]	413.63 ± 39.69 [a]	380.31 ± 31.76 [a,b]
**16:1n7**	26.71 ± 5.23	30.80 ± 6.43	35.90 ± 5.49 [a]	28.60 ± 2.55 [b]
**18:0**	97.99 ± 10.60	126.61 ± 12.56 [a]	146.67 ± 10.99 [a]	167.47 ± 19.87 [a,b]
**18:1n9**	175.30 ± 20.16	225.88 ± 26.57 [a]	713.96 ± 61.80 [a]	667.55 ± 39.57 [a]
**20:0**	1.94 ± 0.14	2.77 ± 0.42 [a]	2.23 ± 0.33 [a]	2.76 ± 0.35 [a,b]
**22:0**	1.32 ± 0.18	2.06 ± 0.36 [a]	1.66 ± 0.35 [a]	1.61 ± 0.32 [a]
**24:0**	4.01 ± 0.83	6.24 ± 0.75 [a]	6.19 ± 0.94 [a]	5.27 ±0.97 [a]
**24:1n9**	1.04 ± 0.23	1.35 ± 0.24 [a]	1.28 ± 0.32	1.05 ± 0.27

The data are expressed as mean values ± SD, n = 10 in each group. [a]p < 0.05 indicates a significant difference: the control group vs. the examined group in the red gastrocnemius muscle; [b]p < 0.05 indicates a significant difference: HFD vs. HFD + CBD in the red gastrocnemius muscle. 14:0 (myristic acid); 16:0 (palmitic acid); 16:1n7 (palmitoleic acid); 18:0 (stearic acid); 18:1n9 (oleic acid); 20:0 (arachidic acid); 22:0 (behenic acid); 24:0 (lignoceric acid); 24:1n9 (nervonic acid).

The content of polyunsaturated fatty acids (PUFA): 18:2n6 (linoleic acid), 18:3n3 (α-linolenic acid), 20:4n6 (arachidonic acid), 20:5n3 (eicosapentaenoic acid) and 20:6n3 (docosahexaenoic acid) in the FFA fraction in the red gastrocnemius muscle was published in our previous publication: Bielawiec et al. (doi: 10.3390/nu13051603).

Supplementary Table S6. Individual fatty acids content in the DAG fraction (nmol/g of wet tissue) in the red gastrocnemius muscle in the control (standard diet) and high-fat diet (HFD) groups after two-week cannabidiol (CBD) treatment.

Fatty acid	Control	CBD	HFD	HFD + CBD
**14:0**	29.93 ± 12.64	30.67 ± 7.10	42.33 ± 16.52	31.87 ± 8.89
**16:0**	214.52 ± 48.79	213.12 ± 14.52	273.88 ± 39.69 [a]	233.31 ± 10.56 [b]
**16:1n7**	7.29 ± 1.56	7.94 ± 1.23	8.72 ± 0.79 [a]	8.46 ± 0.67
**18:0**	75.28 ± 8.65	95.68 ± 8.14 [a]	115.72 ± 10.72 [a]	138.97 ± 10.58 [a,b]
**18:1n9**	55.14 ± 16.18	49.84 ± 5.08	215.66 ± 31.77 [a]	183.39 ± [a,b]
**20:0**	1.83 ± 0.42	2.11 ± 0.16	2.61 ± 0.62 [a]	2.60 ± 0.56 [a]
**22:0**	1.31 ± 0.24	1.41 ± 0.23	1.59 ± 0.43	1.77 ± 0.51
**24:0**	2.02 ± 0.16	2.39 ± 0.34 [a]	2.32 ± 0.76	2.91 ± 0.84 [a]
**24:1n9**	0.60 ± 0.19	0.95 ± 0.25 [a]	0.95 ± 0.33 [a]	1.01 ± 0.23 [b]

The data are expressed as mean values ± SD, n = 10 in each group. $^{a}p < 0.05$ indicates a significant difference: the control group vs. the examined group in the red gastrocnemius muscle; $^{b}p < 0.05$ indicates a significant difference: HFD vs. HFD + CBD in the red gastrocnemius muscle. 14:0 (myristic acid); 16:0 (palmitic acid); 16:1n7 (palmitoleic acid); 18:0 (stearic acid); 18:1n9 (oleic acid); 20:0 (arachidic acid); 22:0 (behenic acid); 24:0 (lignoceric acid); 24:1n9 (nervonic acid).

The content of polyunsaturated fatty acids (PUFA): 18:2n6 (linoleic acid), 18:3n3 (α-linolenic acid), 20:4n6 (arachidonic acid), 20:5n3 (eicosapentaenoic acid) and 20:6n3 (docosahexaenoic acid) in the FFA fraction in the red gastrocnemius muscle was published in our previous publication: Bielawiec et al. (doi: 10.3390/nu13051603).

Supplementary Table S7. Individual fatty acids content in the TAG fraction (nmol/g of wet tissue) in the red gastrocnemius muscle in the control (standard diet) and high-fat diet (HFD) groups after two-week cannabidiol (CBD) treatment.

Fatty acid	Control	CBD	HFD	HFD + CBD
**14:0**	64.68 ± 24.96	87.88 ± 19.25 [a]	175.62 ± 63.99 [a]	109.04 ± 20.32 [a,b]
**16:0**	985.04 ± 518.52	1148.96 ± 170.47	2402.32 ± 571.72 [a]	2243.00 ± 417.58 [a]
**16:1n7**	229.87 ± 177.07	148.85 ± 29.75	144.92 ± 88.65	95.62 ± 41.97 [a]
**18:0**	204.15 ± 98.17	171.40 ± 12.28	819.29 ± 224.85 [a]	687.64 ± 88.36 [a]
**18:1n9**	383.66 ± 218.08	563.89 ± 149.13	1898.44 ± 520.64 [a]	1916.44 ± 496.42 [a]
**20:0**	4.86 ± 1.40	4.42 ± 1.16	11.16 ± 2.54 [a]	12.67 ± 2.06 [a]
**22:0**	6.30 ± 1.17	6.17 ± 2.00	8.12 ± 2.04 [a]	8.73 ± 1.78 [a]
**24:0**	3.21 ± 0.80	3.26 ± 1.32	3.15 ± 1.46	3.15 ± 0.74
**24:1n9**	3.76 ± 0.64	3.07 ± 1.08	3.07 ± 0.85	4.31 ± 1.23 [b]

The data are expressed as mean values ± SD, n = 10 in each group. $^{a}p < 0.05$ indicates a significant difference: the control group vs. the examined group in the red gastrocnemius muscle; $^{b}p < 0.05$ indicates a significant difference: HFD vs. HFD + CBD in the red gastrocnemius muscle. 14:0 (myristic acid); 16:0 (palmitic acid); 16:1n7 (palmitoleic acid); 18:0 (stearic acid); 18:1n9 (oleic acid); 20:0 (arachidic acid); 22:0 (behenic acid); 24:0 (lignoceric acid); 24:1n9 (nervonic acid).

The content of polyunsaturated fatty acids (PUFA): 18:2n6 (linoleic acid), 18:3n3 (α-linolenic acid), 20:4n6 (arachidonic acid), 20:5n3 (eicosapentaenoic acid) and 20:6n3 (docosahexaenoic

acid) in the FFA fraction in the red gastrocnemius muscle was published in our previous publication: Bielawiec et al. (doi: 10.3390/nu13051603).

Supplementary Table S8. Individual fatty acids content in the PL fraction (nmol/g of wet tissue) in the red gastrocnemius muscle in the control (standard diet) and high-fat diet (HFD) groups after two-week cannabidiol (CBD) treatment.

Fatty acid	Control	CBD	HFD	HFD + CBD
**14:0**	79.31 ± 12.92	73.20 ± 9.16	77.62 ± 12.21	79.21 ± 5.08
**16:0**	6170.28 ± 362.27	6367.10 ± 326.43	6177.77 ± 602.57	7524.57 ± 254.85 [a,b]
**16:1n7**	155.33 ± 16.02	174.84 ± 23.24 [a]	106.52 ± 6.73 [a]	121.46 ± 8.13 [a,b]
**18:0**	5028.22 ± 440.77	5556.33 ± 236.17 [a]	6338.56 ± 179.13 [a]	7599.20 ± 464.05 [a,b]
**18:1n9**	919.62 ± 60.63	892.17 ± 74.69	1377.28 ± 69.71 [a]	1705.48 ± 107.44 [a,b]
**20:0**	14.51 ± 1.32	17.20 ± 1.55 [a]	15.04 ± 2.75	15.39 ± 1.45
**22:0**	15.90 ± 2.48	19.10 ± 3.77 [a]	15.76 ± 1.66	16.93 ± 1.94
**24:0**	12.94 ± 3.60	13.97 ± 3.09	16.05 ± 4.15	16.77 ± 2.79 [a]
**24:1n9**	20.79 ± 1.76	12.38 ± 1.60 [a]	17.25 ± 2.99 [a]	15.11 ± 2.35 [a]

The data are expressed as mean values ± SD, n = 10 in each group. [a]$p < 0.05$ indicates a significant difference: the control group vs. the examined group in the red gastrocnemius muscle; [b]$p < 0.05$ indicates a significant difference: HFD vs. HFD + CBD in the red gastrocnemius muscle. 14:0 (myristic acid); 16:0 (palmitic acid); 16:1n7 (palmitoleic acid); 18:0 (stearic acid); 18:1n9 (oleic acid); 20:0 (arachidic acid); 22:0 (behenic acid); 24:0 (lignoceric acid); 24:1n9 (nervonic acid).

The content of polyunsaturated fatty acids (PUFA): 18:2n6 (linoleic acid), 18:3n3 (α-linolenic acid), 20:4n6 (arachidonic acid), 20:5n3 (eicosapentaenoic acid) and 20:6n3 (docosahexaenoic acid) in the FFA fraction in the red gastrocnemius muscle was published in our previous publication: Bielawiec et al. (doi: 10.3390/nu13051603).
