# Supplementary material for: Cannabidiol improves muscular lipid profile by affecting the expression of fatty acid transporters and inhibiting de novo lipogenesis
Source: Sci Rep. 2023 Mar 6;13:3694. doi: 10.1038/s41598-023-30872-w (PMC9988888; doi:10.1038/s41598-023-30872-w)
Supplement: Supplementary file 2 — Supplementary Information 2. [file 41598_2023_30872_MOESM2_ESM.pdf]

Loading order in all Western blots: Control, HFD, CBD, HFD+CBD.

On the left are stain-free gels showing the total protein loading used for normalization.

On the right are corresponding membranes with the detected proteins.

White skeletal muscle

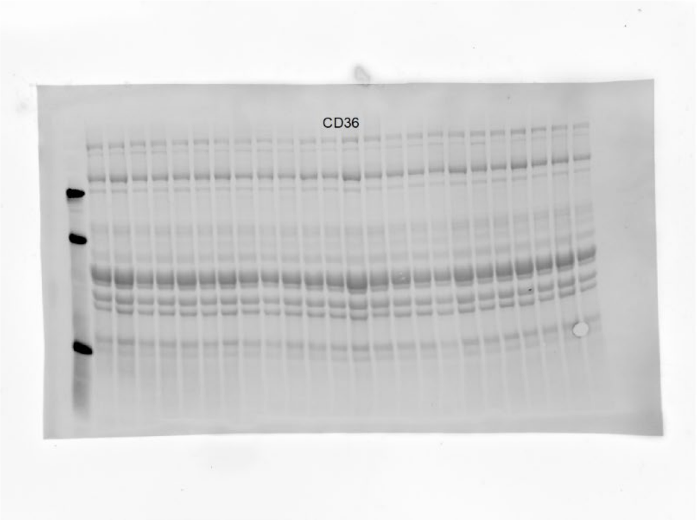

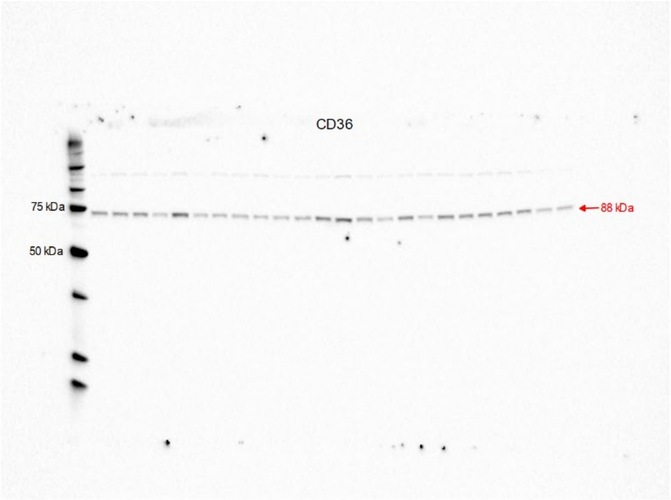

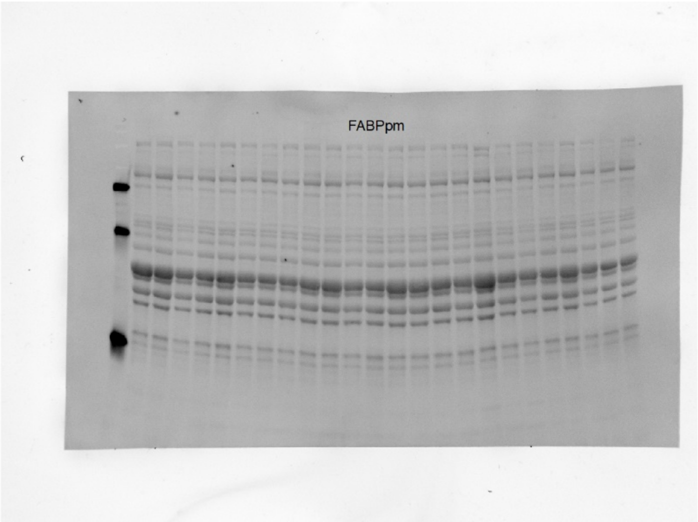
FABPpm

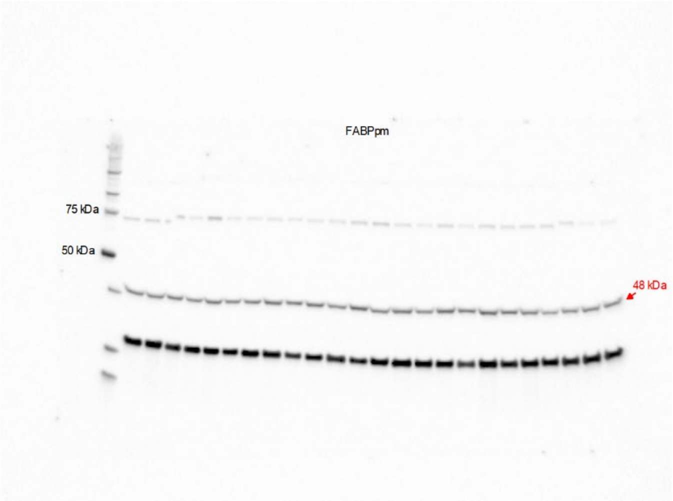
FABPpm
75 kDa
50 kDa
48 kDa

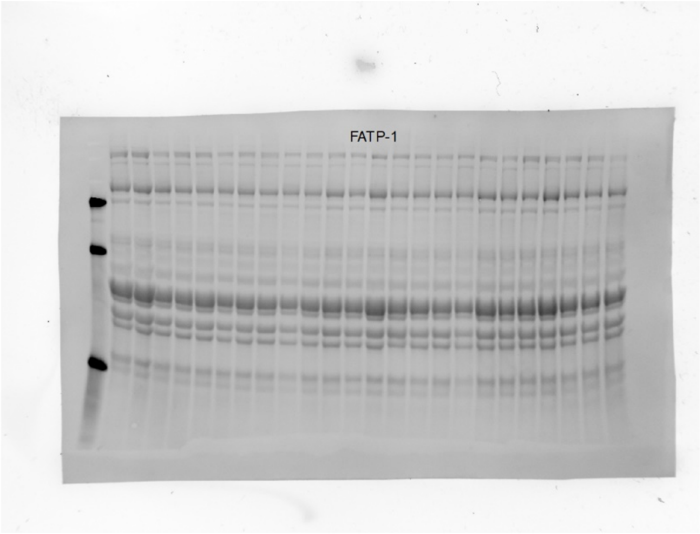
FATP-1

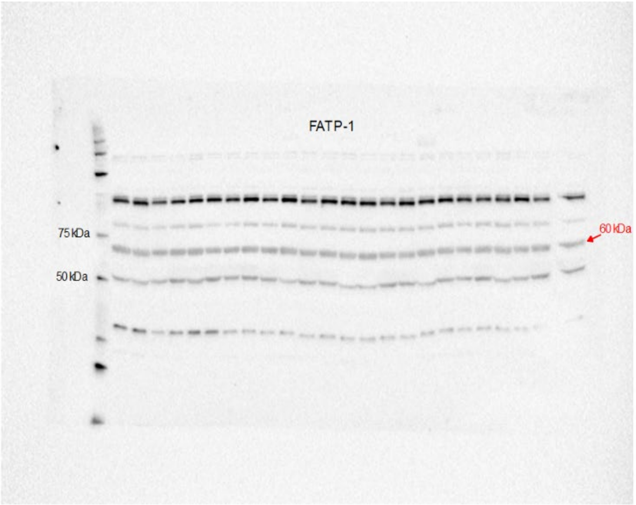
FATP-1
75 kDa
50 kDa
60 kDa

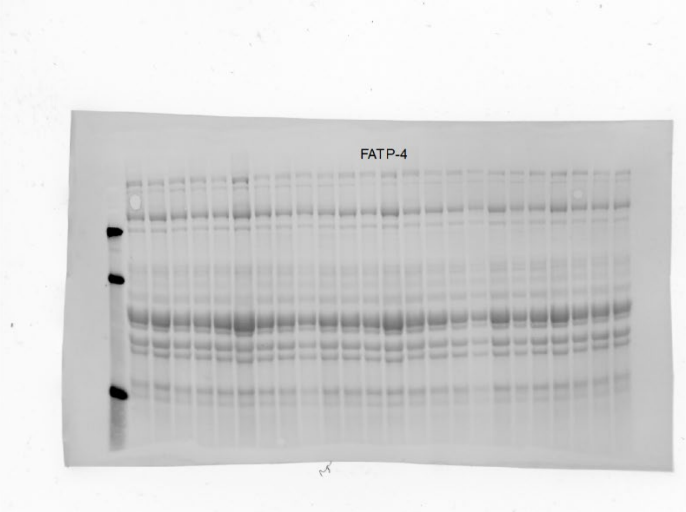
FATP-4

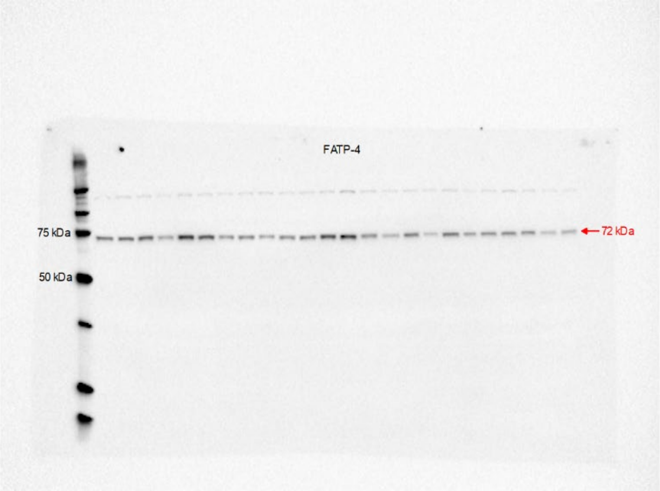
FATP-4
75 kDa
50 kDa
72 kDa

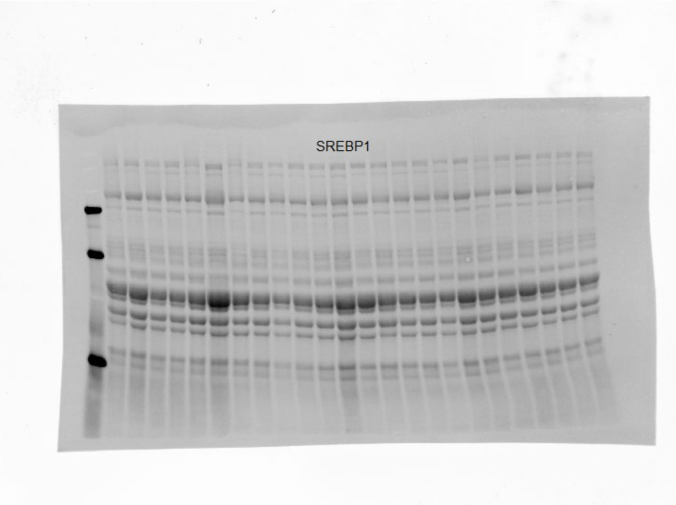
SREBP1

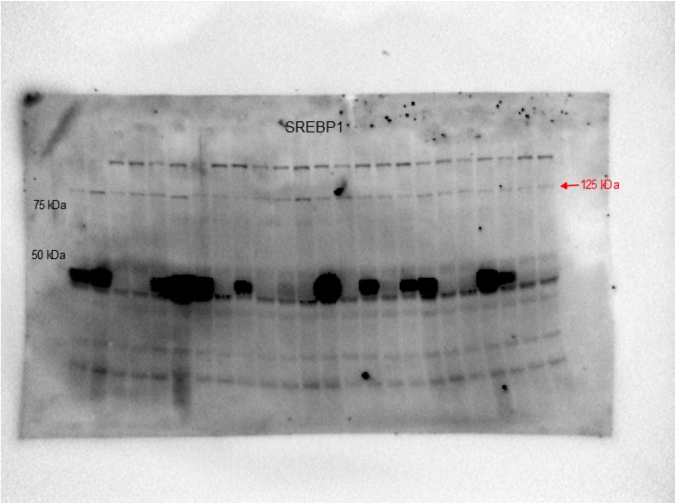
SREBP1
75 kDa
50 kDa
125 kDa

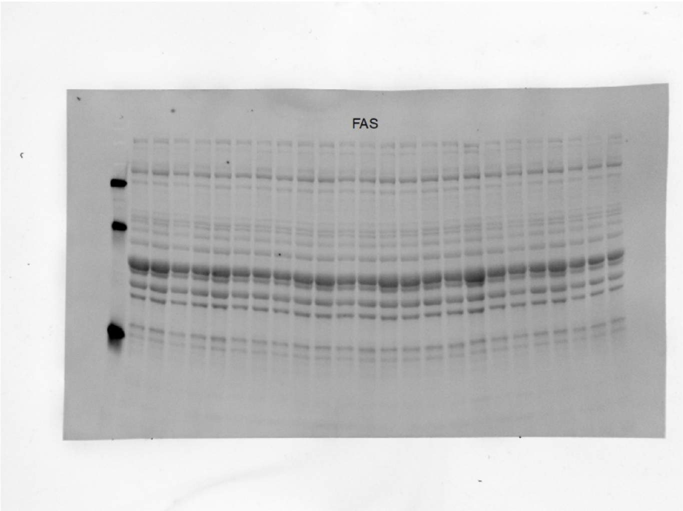
FAS

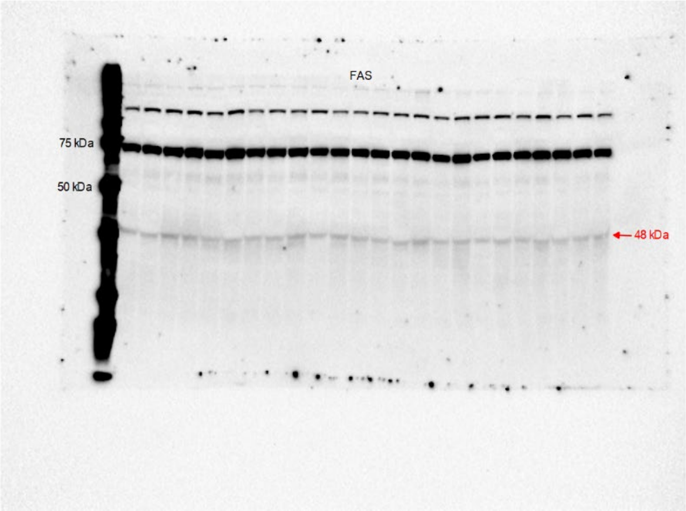
FAS
75 kDa
50 kDa
48 kDa

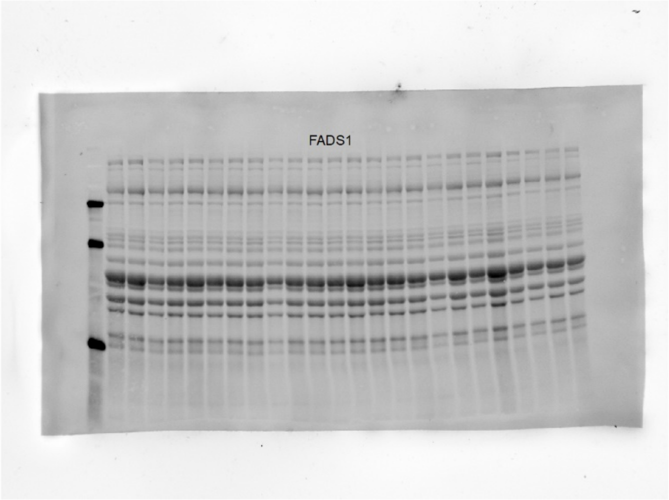
FADS1

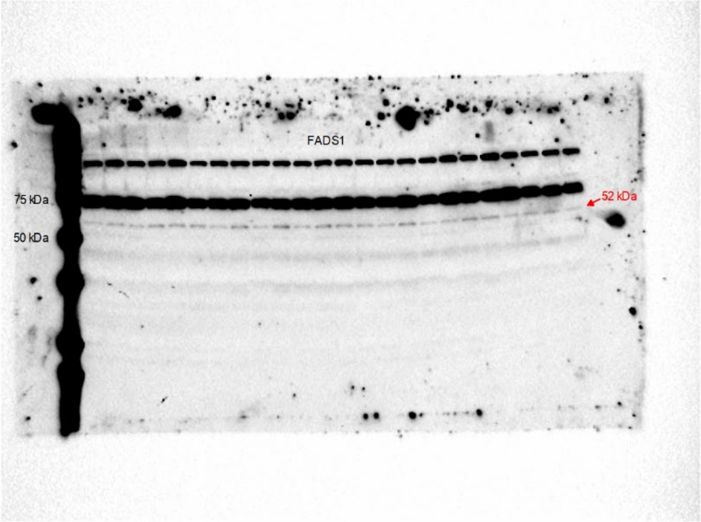
FADS1
75 kDa
50 kDa
52 kDa

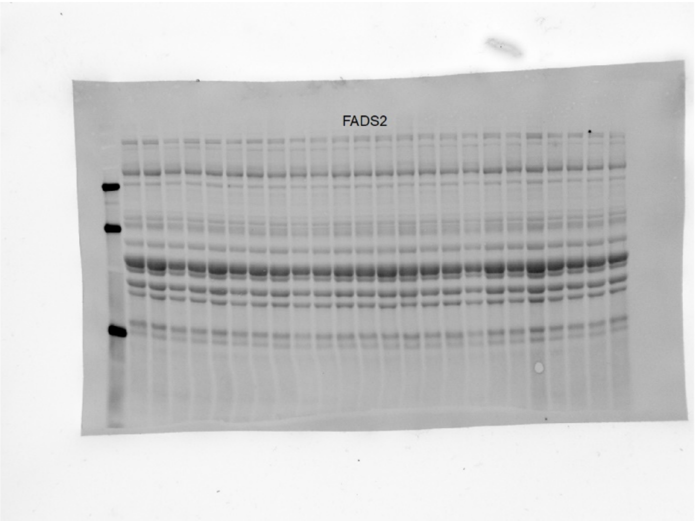
FADS2

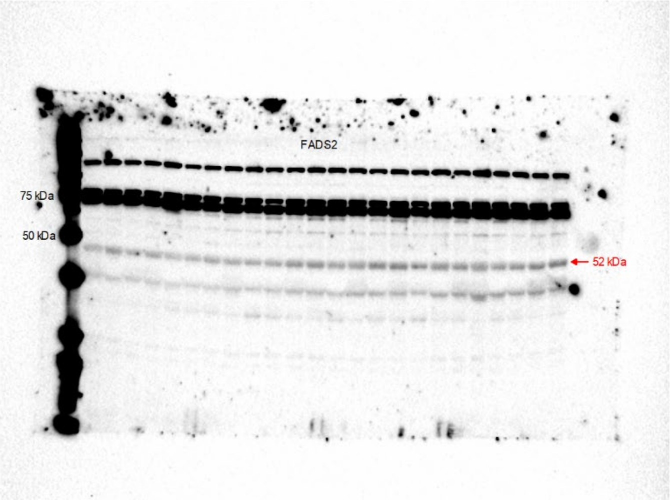
FADS2
75 kDa
50 kDa
52 kDa

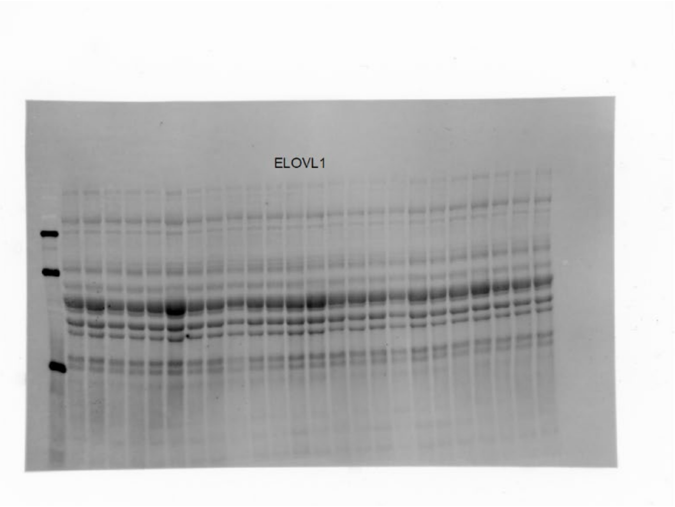
ELOVL1

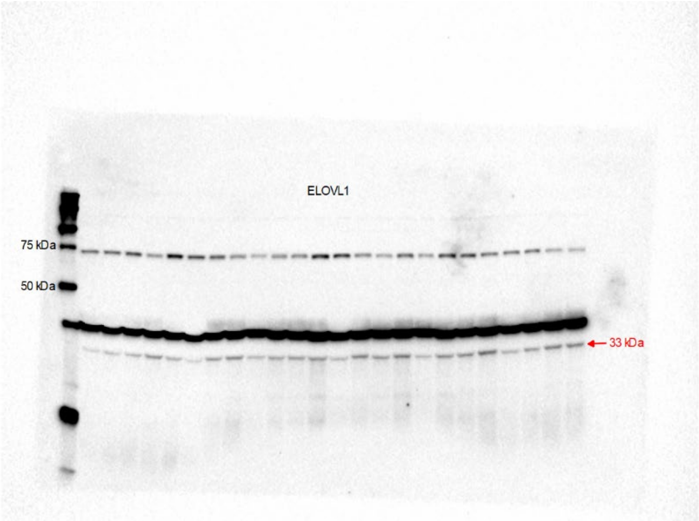
ELOVL1
75 kDa
50 kDa
33 kDa

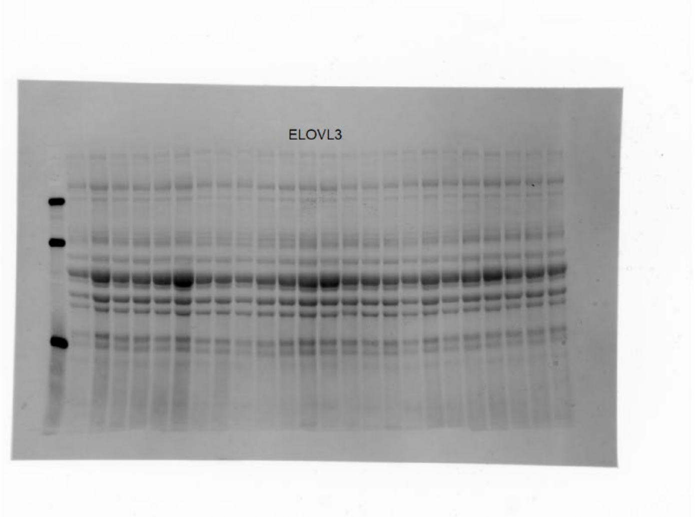
ELOVL3

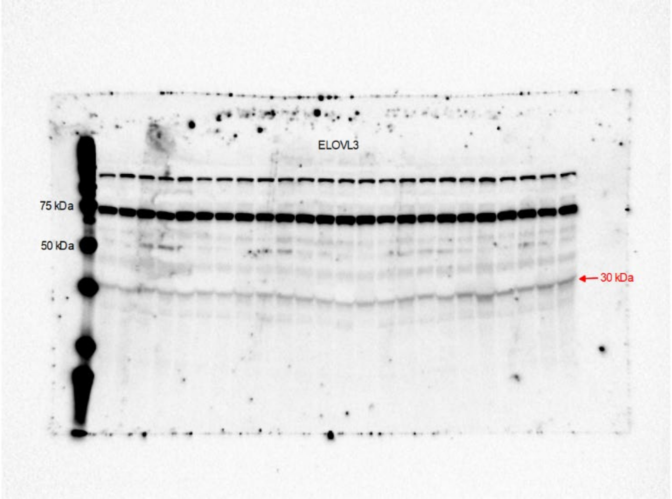
ELOVL3
75 kDa
50 kDa
30 kDa

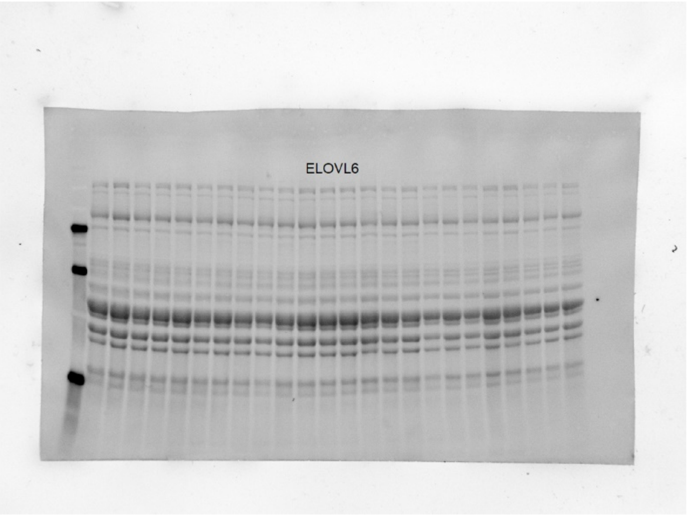
ELOVL6

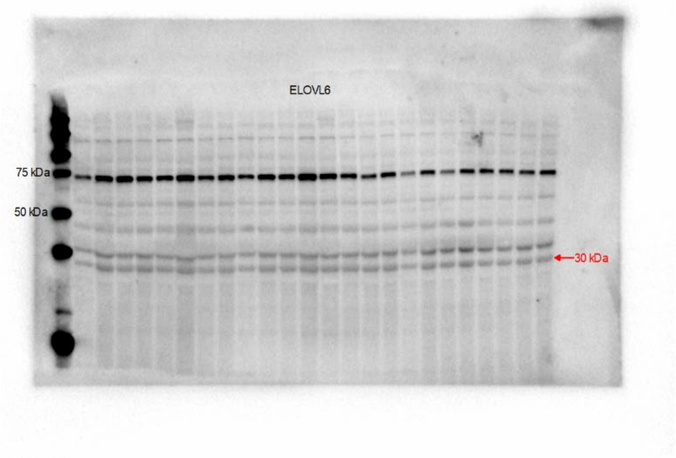
ELOVL6
75 kDa
50 kDa
30 kDa

Red skeletal muscle

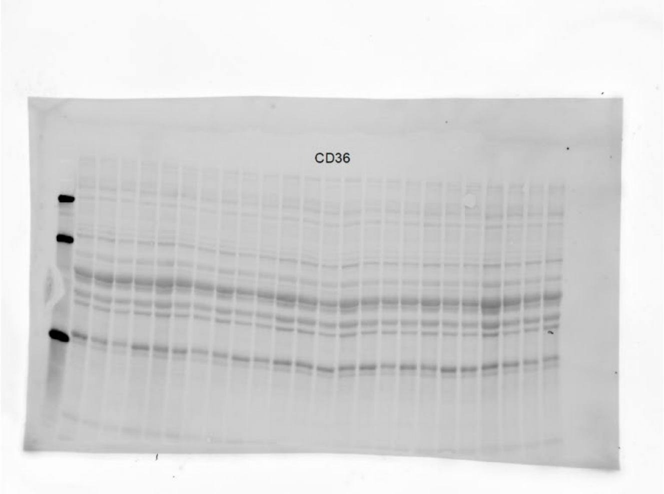

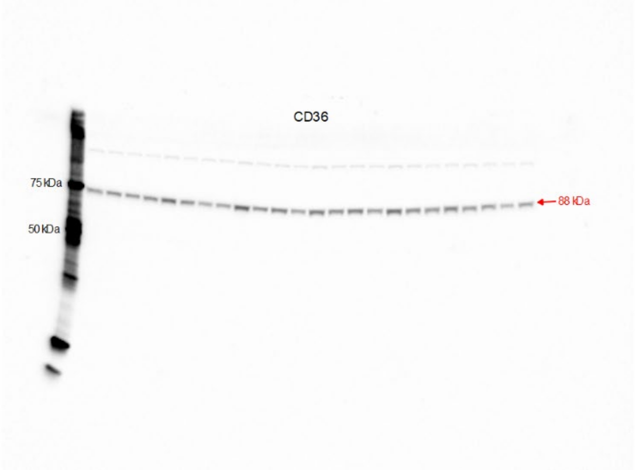

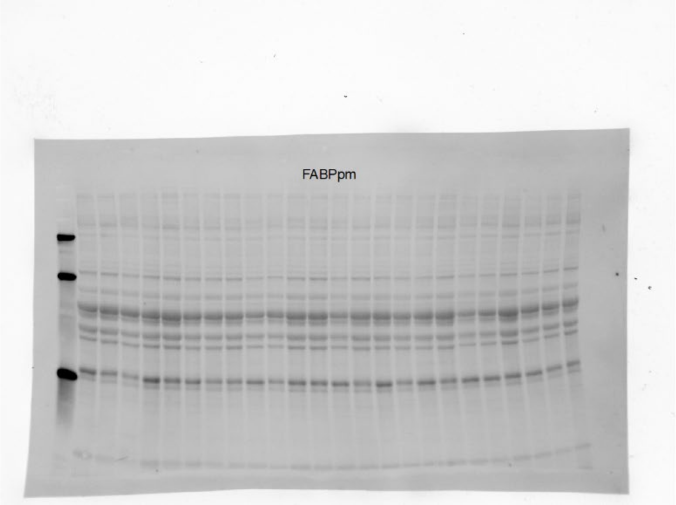

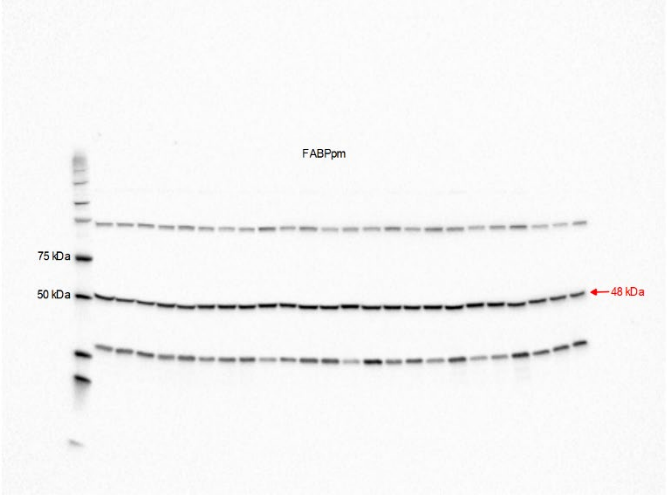

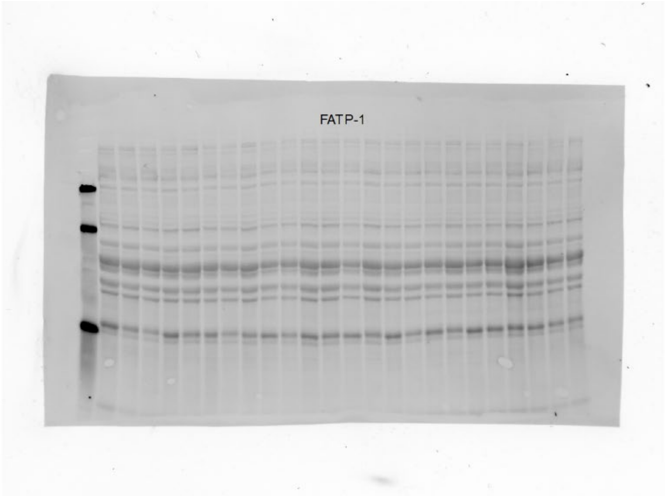
FATP-1

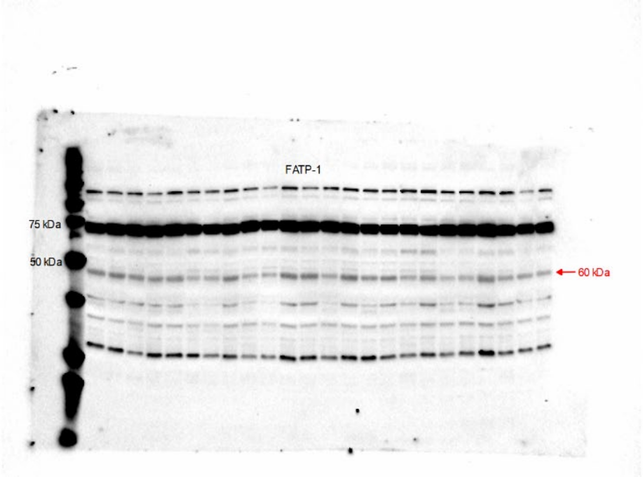
FATP-1
75 kDa
50 kDa
60 kDa

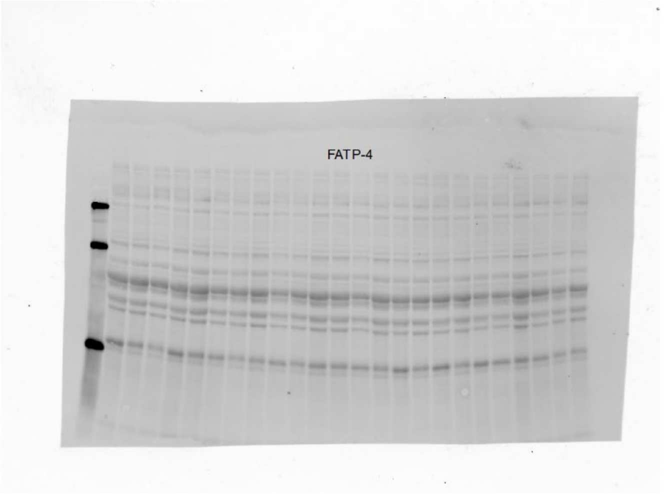
FATP-4

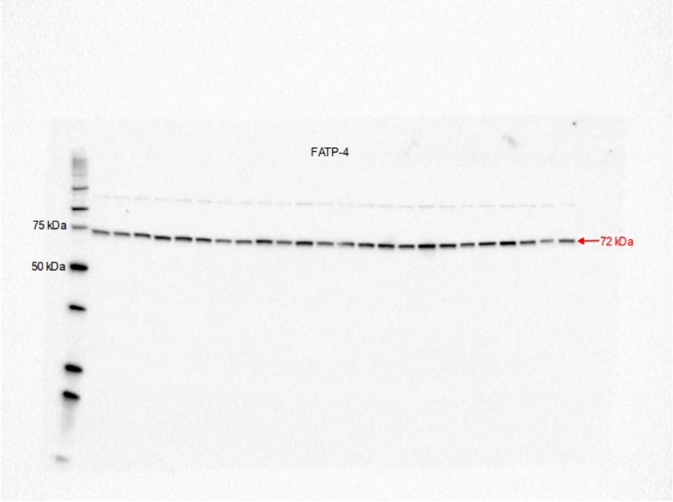
FATP-4
75 kDa
50 kDa
72 kDa

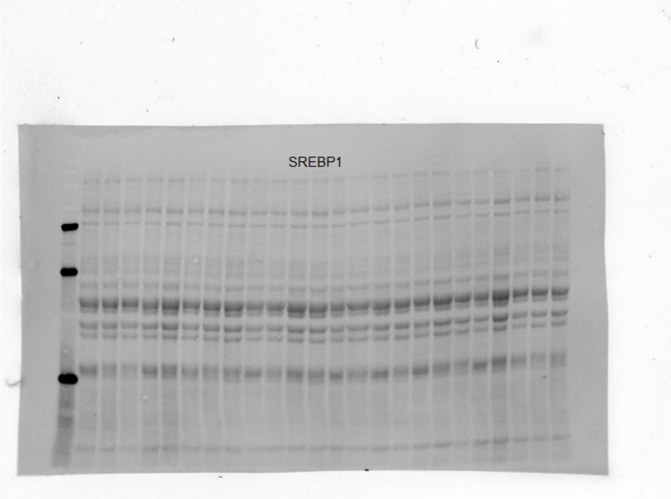
SREBP1

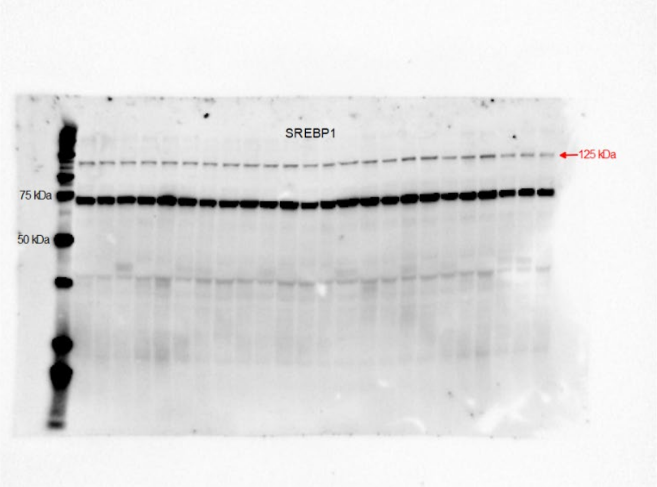
SREBP1
125 kDa
75 kDa
50 kDa

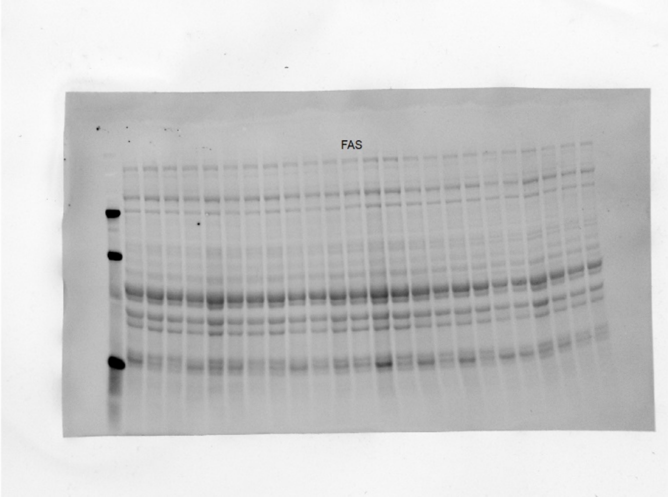
FAS

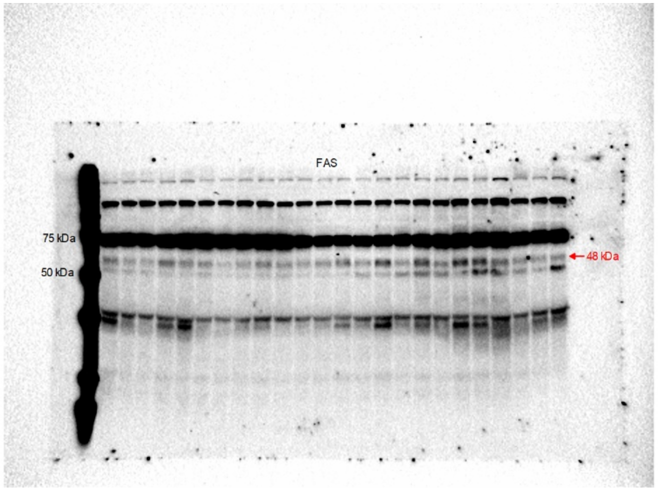
FAS
75 kDa
48 kDa
50 kDa

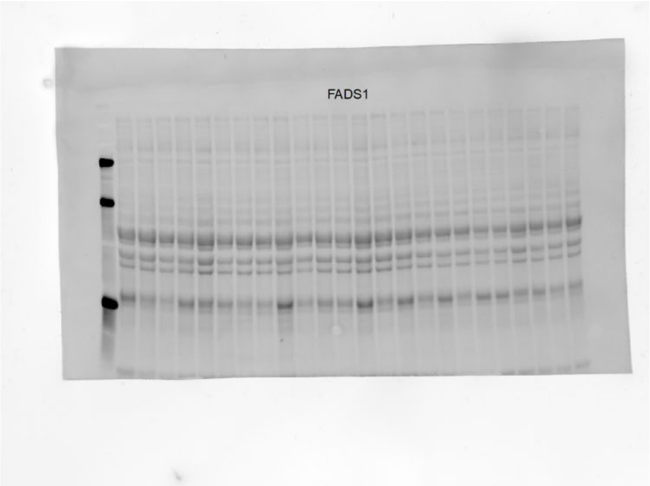
FADS1

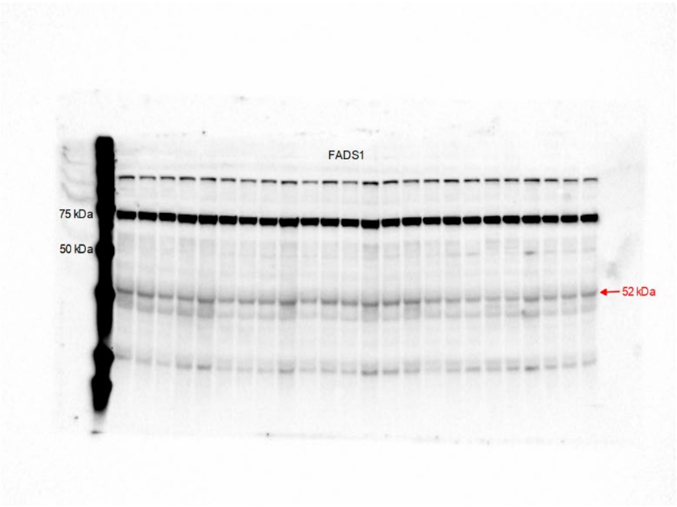
FADS1
75 kDa
50 kDa
52 kDa

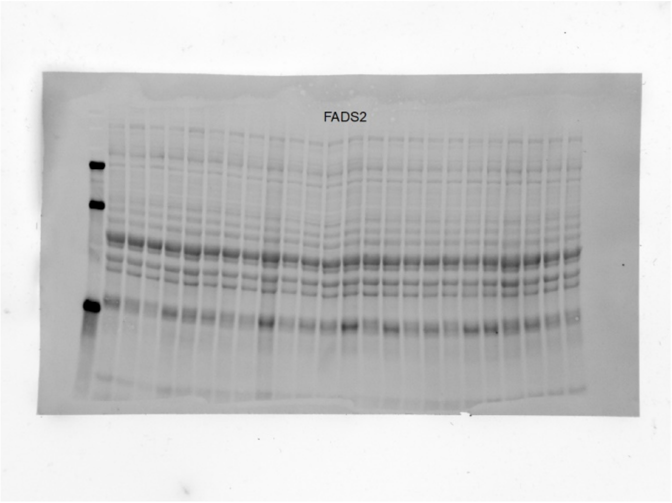
FADS2

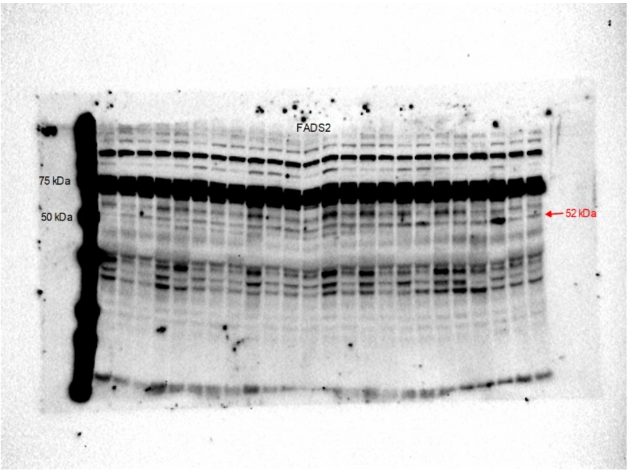
FADS2
75 kDa
50 kDa
52 kDa

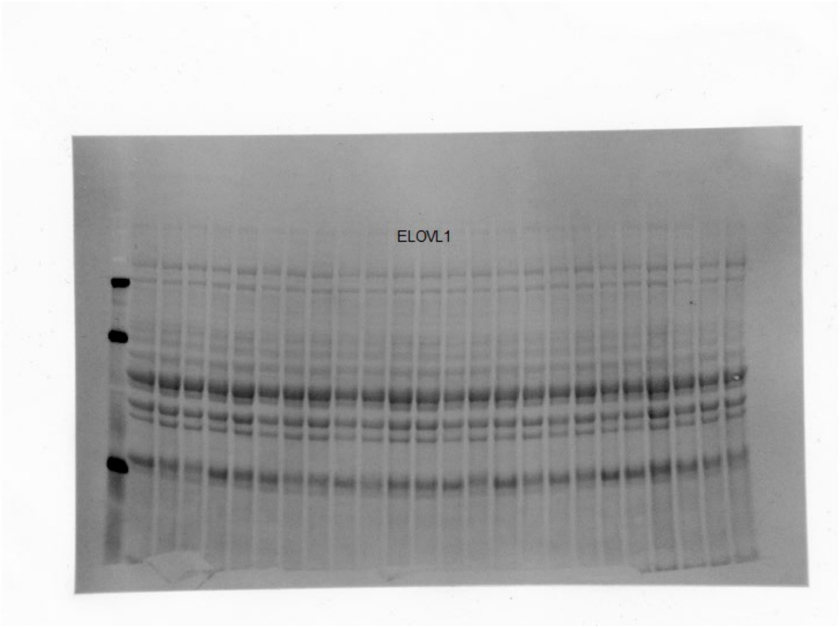
ELOVL1

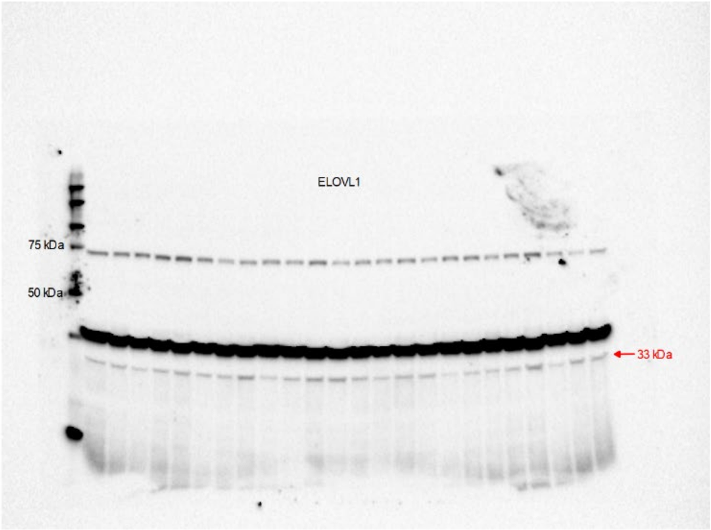
ELOVL1
75 kDa
50 kDa
33 kDa

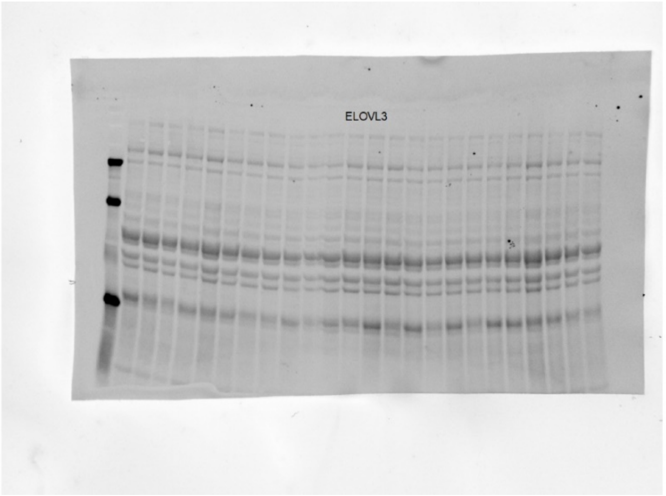
ELOVL3

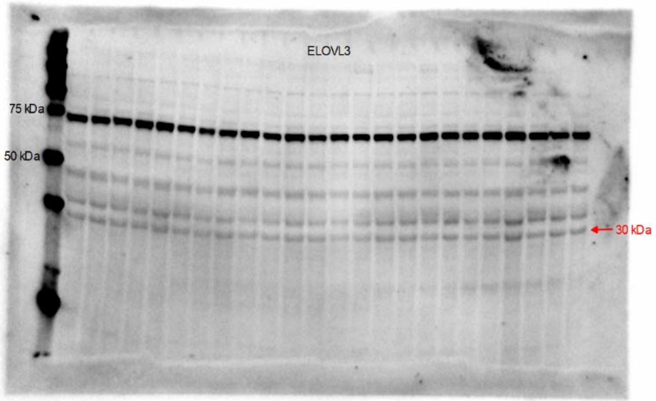
ELOVL3
75 kDa
50 kDa
30 kDa

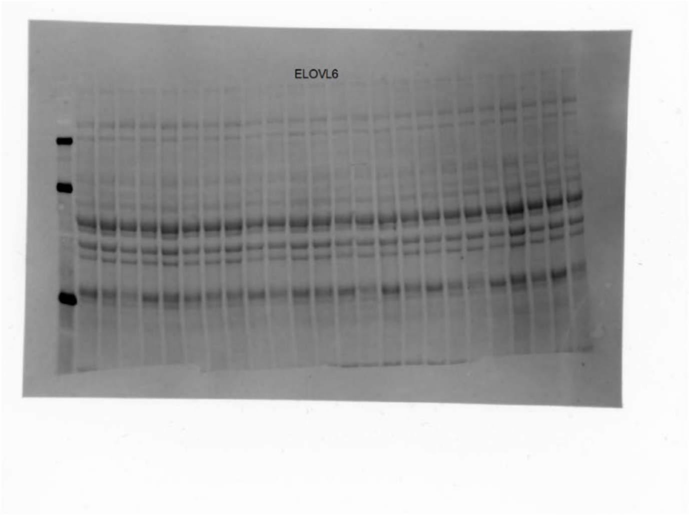
ELOVL6

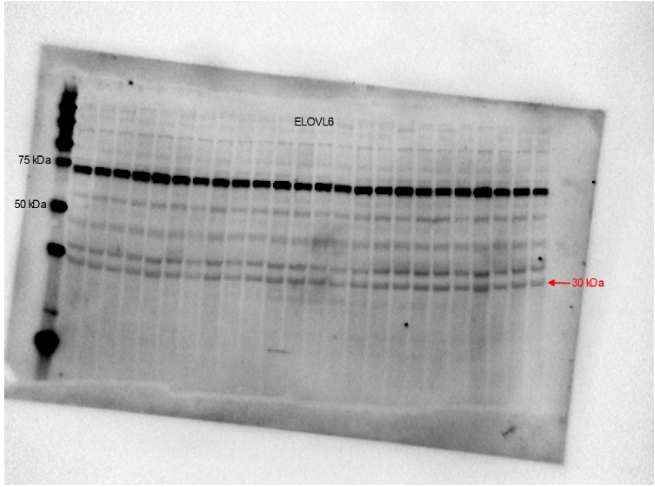
ELOVL6
75 kDa
50 kDa
30 kDa
